# Diagnostic accuracy of SeptiCyte® RAPID to discriminate sepsis from non-infectious critical illness in patients meeting sepsis criteria according to sepsis-3 definition at ICU admission

**DOI:** 10.1007/s10096-026-05431-6

**Published:** 2026-02-23

**Authors:** María Luisa Cantón-Bulnes, José Luís García-Garmendia, Ángel Estella, Adela Fernández-Galilea, Isidro Blanco, María Antonia Estecha-Foncea, Marina Gordillo-Resina, Jorge Rodríguez-Gómez, Juan Jesús Pineda-Capitán, Carmen Martínez-Fernández, Ana Escoresca-Ortega, Rosario Amaya-Villar, Juan Mora-Ordóñez, Sara González-Soto, José Garnacho-Montero

**Affiliations:** 1 Hospital Universitario Virgen Macarena (Sevilla), Intensive Care Unit, Seville, Spain; 2 Hospital San Juan de Dios del Aljarafe (Sevilla), Intensive Care Unit, Sevilla, Spain; 3 HospitalUniversitario de Jerez de la Frontera (Cádiz), Intensive Care Unit, Cadiz, Spain; 4 Medicine and Surgery Department, University of Cadiz, INIBiCA, Spain, España; 5 HospitalUniversitario Virgen de la Victoria (Málaga), Intensive Care Unit, Málaga, Spain; 6 HospitalUniversitario Reina Sofía (Córdoba), Intensive Care Unit, Córdoba, Spain; 7 HospitalUniversitario Virgen del Rocío (Sevilla), Intensive Care Unit, Sevilla, Spain; 8 HospitalUniversitario Regional de Málaga (Málaga), Intensive Care Unit, Málaga, Spain

**Keywords:** ICU, Sepsis, Septycite, Mortality, Biomarkers, Diagnosis accuracy

## Abstract

**Objectives:**

The aim of this study was to validate the SeptiCyte® RAPID assay, a molecular test to distinguish sepsis from sterile inflammation, by determining its diagnostic accuracy in critically ill patients who meet criteria for sepsis according to Sepsis-3 definition on ICU admission

**Design:**

This is an observational, prospective, and multicenter study.Setting: Carried out in seven hospitals in Andalusia (Spain). A 2.5 mL whole blood sample was collected and tested in a SeptiCyte RAPID kit on a real time PCR platform (IdyllaTM). A score from 0 to 15 (SeptiScore™) was generated that falls into four bands based on the increasing likelihood of infection-positive systemic inflammation.

**Patients:**

Patients aged 18 years or older, admitted to the ICU with a diagnosis of sepsis.Main results: We enrolled 354 patients, of whom 86 (24.3%) did not present sepsis at the researchers´ discretion. SeptiCyte® RAPID showed an AUC of [0.84 (CI95% 0.79-0.87)] for differentiating sepsis from sterile systemic inflammation. SeptiCyte® RAPID was significantly better for sepsis diagnosis than CRP [0.75 (CI95% 0.70-0.80)] (p =0.003) but without significant differences with PCT [0.80 (CI95% 0.75-0.84)]. SeptiScore distribution in patients with sepsis was higher than patients with sterile inflammation, with a PPV of 68.8% and 92.2% (Bands 3 and 4) for sepsis diagnosis and a PPV of 100% for sterile inflammation (Band 1). Independent risk factors for sepsis were estimated probability of sepsis [OR 8.02 (CI 95% 4.50-14.28), p<0.001], SeptiScore [OR 1.64 (CI 95% 1.35-1.99), p<0.001], and log procalcitonin [OR 1.68 (CI 95% 1.09-2.59), p=0.020].Conclusions: SeptiCyte® RAPID discriminates sepsis from sterile inflammation in critically ill adults, adding value to the diagnosis of sepsis.

**Purpose:**

The aim of this study was to validate the SeptiCyte® RAPID assay, a molecular test to distinguish sepsis from sterile inflammation, by determining its diagnostic accuracy in critically ill patients who meet criteria for sepsis according to Sepsis-3 definition on ICU admission.Methods: This is an observational, prospective, and multicenter study. Carried out in seven hospitals in Andalusia (Spain). A 2.5 mL whole blood sample was collected and tested in a SeptiCyte RAPID kit on a real time PCR platform (IdyllaTM). A score from 0 to 15 (SeptiScore™) was generated that falls into four bands based on the increasing likelihood of infection-positive systemic inflammation.Patients aged 18 years or older, admitted to the ICU with a diagnosis of sepsis were included.

**Results:**

We enrolled 353 patients, of whom 86 (23.7%) did not present sepsis at the researchers´ discretion. SeptiCyte® RAPID showed an AUC of [0.84 (CI95% 0.79-0.87)] for differentiating sepsis from sterile systemic inflammation. SeptiCyte® RAPID was significantly better for sepsis diagnosis than CRP [0.75 (CI95% 0.70-0.80)] (p =0.003) but without significant differences with PCT [0.80 (CI95% 0.75-0.84)]. SeptiScore distribution in patients with sepsis was higher than patients with sterile inflammation, with a PPV of 68.8% and 92.2% (Bands 3 and 4) for sepsis diagnosis and a PPV of 100% for sterile inflammation (Band 1). Independent risk factors for sepsis were physicians´ subjective likelihood of sepsis [intermediate probabilty OR 4.55 (95% CI 1.61-12.83), p=0.004 and high probabilty OR 72.04 (95% CI 20.85-248.93), p<0.001], SeptiScore [OR 1.65 (95% CI 1.32-2.07), p<0.001], and log procalcitonin [OR 1.74 ( 95% CI 1.08-2.79), p=0.022].Conclusions: SeptiCyte® RAPID discriminates sepsis from sterile inflammation in critically ill adults, adding value to the diagnosis of sepsis.

**Supplementary Information:**

The online version contains supplementary material available at 10.1007/s10096-026-05431-6.

## Introduction

The incidence of sepsis and septic shock is increasing worldwide with a high associated mortality. Early identification of sepsis and the implementation of treatment bundles have been shown to improve outcomes in patients with sepsis and septic shock [1]. However, early diagnosis of sepsis can be challenging in many cases. In the initial stages, patients may present with clinical signs similar to many other non-infectious diseases [2]. Klouwenberg et al. reported that the clinical diagnosis of sepsis on admission to the ICU correlated poorly with the presence of infection in a *post hoc* evaluation [3].

Early diagnosis of sepsis can be challenging. In fact, sepsis lacks a diagnostic gold standard, with blood cultures being negative in 50–70% of the cases [4, 5]. In such circumstances, unnecessary administration of broad-spectrum antibiotics for suspected sepsis may negatively contribute to the growing problem of multidrug-resistant microorganisms and increase the risk of adverse drug reactions [6]. Several biomarkers, specially C-reactive protein (CRP) and procalcitonin (PCT) have been evaluated for their diagnostic ability to distinguish infection from non-infectious critical illness [7]. Despite the fact that these biomarkers correlate with the presence of systemic inflammation, they cannot reliably discriminate sepsis from other non-infectious causes of systemic inflammatory response [8, 9].

Due to the biological complexity of sepsis, it has been postulated that a strategy based on a panel of multiple biomarkers such as genes expression signatures, may be more appropriate for diagnostic purposes than a biomarker-based stratification tool [10]. While transcriptome sequencing of messenger ribonucleic acid (mRNA) may in the future serve as a useful diagnostic tool for differentiating between infection and non-infectious critical illness, the currently available SeptiCyte^®^ LAB measures the expression of four host-response mRNAs in peripheral blood using reverse transcription quantitative polymerase chain reaction (RT-qPCR), with a turn-around time of approximately 6 h. Several clinical studies have validated this test in critically ill patients meeting the criteria for systemic inflammatory response syndrome (SIRS) [10–12].

A new test, the SeptiCyte^®^ RAPID assay with a 1 h turn-around time was recently launched. SeptiCyte^®^ RAPID is a gene expression assay that uses RT-qPCR to measure the relative expression levels of two host response genes, phospholipase A2 (PLA2G7) and placenta specific 8 (PLAC8), which are both indicative of a dysregulated immune response during sepsis. SeptiCyte^®^ RAPID generates a quantitative score (SeptiScore^®^), which increases as the likelihood of sepsis increases. A strong upregulation of these genes involved in immune function regulation (PLAC8) and inflammatory signaling (PLA2G7) occurs early in patients with infections and may differetiate sepsis from non-infectious inflammation [13–15].

To our knowledge, the SeptiCyte^®^ RAPID assay has not been prospectively validated in patients with suspicion of sepsis according to the new Sepsis-3 criteria [16]. The aim of this study was to validate the SeptiCyte^®^ RAPID assay by determining its diagnostic accuracy in critically ill patients meeting the new Sepsis-3 criteria on admission to the ICU. We also compared this simplified SeptiCyte^®^ RAPID assay with CRP and PCT, the biomarkers most widely used for the diagnosis of sepsis.

## Materials and methods

This multicenter prospective cohort study was conducted between March 3, 2022 and December 20, 2022 in seven ICUs in Andalusia (Spain), and coordinated by the Virgen Macarena University Hospital in Seville. However, two hospitals (HUVR and HURM) started on September and October 2022 for organizational problems and delay in the approval by the Research Ethics Committees. The study was not registered in any public clinical trial database, as it was an observational diagnostic accuracy study with no therapeutic intervention; however, the study protocol is provided in the Supplementary Material (Supplementary Digital Content, SDC). The study followed the Standards for Reporting of Diagnostic Accuracy (STARD) guidelines for reporting studies of diagnostic accuracy [17], as is shown in S-Table 1 of SDC.

Patients aged 18 years or older, admitted to the ICU with a diagnosis of sepsis, according to the Sepsis-3 definition [16] were included. Subjects were excluded if they were pregnant, or the clinical picture suggestive of sepsis had started more than 48 h previously. The study was approved by the Research Ethics Committees of the participating hospitals.

**Fig. 1:**
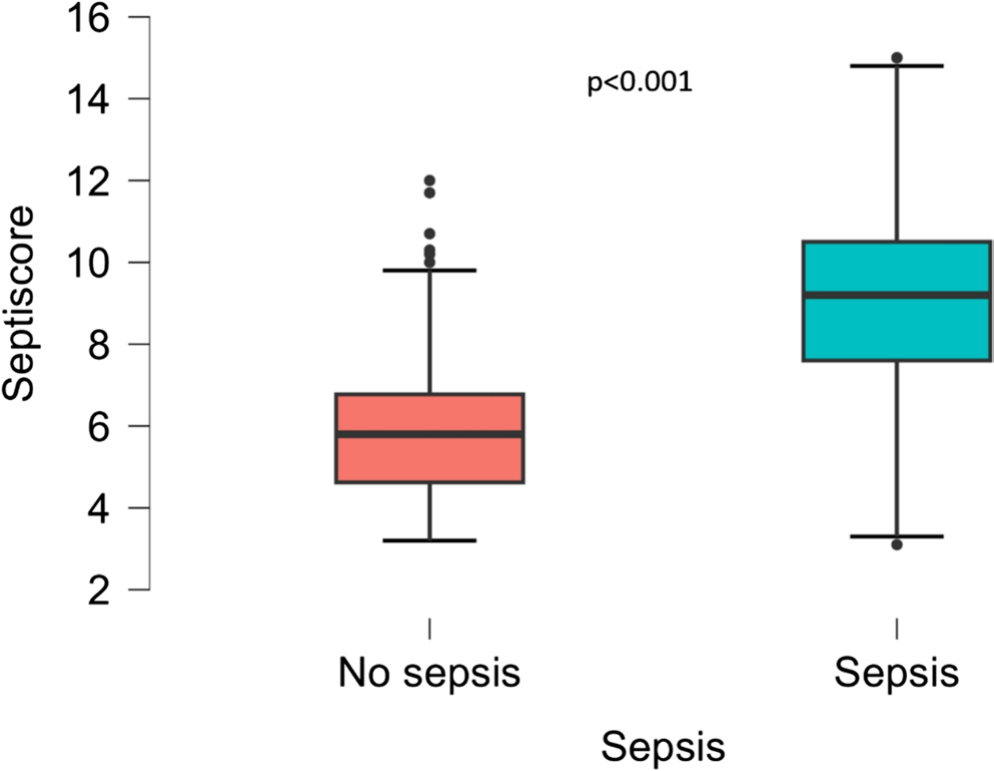
Results of the SeptiScore RAPID for patients with or without infection

Blood samples for the determinations of analytical variables (including C-reactive protein and procalcitonin) and SeptiCyte^®^ were obtained as soon as possible after admission to the ICU when the probable diagnosis of sepsis was established. Analysis to produce the SeptiCyte^®^ RAPID scores (SeptiScore) was performed automatically on the Idylla™ platform (Biocartis NV, Mechelen, Belgium), an approved and CE certified assay, which is capable of fully automated nucleic acid testing, including extraction, amplification, and detection, all in approximately one hour. According to the manufacturer’s specification, the resulting score was finally classified into four probability bands reflecting an increasing sepsis likelihood; scores lesser than or equal to 4.9 represented band 1 and were categorized as “sepsis unlikely,” whereas scores 5–6.1, 6.2–7.3, and greater than 7.4 represented bands 2, 3, and 4, respectively, and were categorized as “sepsis likely” (S-Figure 1). Detailed information about how the samples were processed is included in the SDC.

Written consent from the patient or next of kin was obtained within 48 h of ICU admission and the blood sample was discarded if written consent was not obtained. Since sepsis is a time-dependent process and the clinical outcome depends on how quickly it is recognized, this assay should be performed as soon as possible after clinical suspicion of sepsis. Based on this argument, the research ethics committees allowed blood sample extractions prior to obtaining written permission.

Variables collected included demographic characteristics, comorbidities, type of infection (community-acquired or hospital-acquired illness), and presentation with sepsis or shock [16]. Physiological variables such as body temperature, respiratory rate, heart rate, mean arterial pressure were recorded (the worst values during the first 24 h after ICU admission). Complete blood count and biochemical variables (lactate, urea, creatinine, CRP and PCT) were measured at each center using the first blood sample obtained at study enrollment. Disease severity was assessed using the Acute Physiology and Chronic Health Evaluation (APACHE II) score [18] and the Sequential Organ Failure Assessment (SOFA) scale [19]. For both scores, the worst data from the first 24 h in the ICU were used.

Blood cultures and conventional cultures from the presumed site of infection were obtained from all patients at study entry. Multiplex PCR on blood or respiratory samples were not available at that time in any of the participant hospitals. Positive blood cultures were defined as: positive blood cultures with a clinically relevant organism. Single blood cultures with a skin organism such as coagulase-negative Staphylococci, *Corynebacterium* spp., and *Bacillus* spp. were considered contamination.

The subjective probability of sepsis was estimated at ICU admission by the attending physician blinded to the transcriptome results (low < 30%, intermediate 30–70%, high > 70%), according to the clinical and analytical data. Finally, patients were categorized as “sepsis” or “non-infectious critical illness” by two investigators at each participating site who were aware of the clinical, analytical, and microbiological data according to the Sepsis-3 definition, but blinded to the SeptiCyte^®^ results. In patients with negative microbiology, the likelihood of infection was established by the presence of clinical and analytical features strongly suggestive of infection. Non-infectious critical illness was defined as organ dysfunction as defined by Sepsis-3 definition not related to an infection. The final diagnosis of sepsis was made at ICU discharge. Prior to the start of the study, a meeting was held with all the researchers to standardize criteria regarding the diagnosis. Moreover, the Steering Committee (MLCB, AEG, JLGG, and JGM) reviewed all cases and contacted local investigators in case of doubts. Discussions between the Steering Committee and the attending team served to reach consensus in case of uncertainties.

A standardized form was developed for all prospective data collection using RedCAP software (Research Electronic Data Capture, Vanderbilt University, Nashville, TN). All patients were followed up until death or hospital discharge. All details regarding SeptiCyte^®^ RAPID assay are shown in S-Methods in SDC.

### Statistical analyses

We estimated that the prevalence of confirmed sepsis in patients admitted to the ICU with suspected sepsis was 75% [20]. Given this prevalence, assuming that the clinical assessments have a sensitivity and a specificity of 80%, and for the SeptiCyte^®^ RAPID test a sensitivity and a specificity of 95%, we estimated that with a confidence level of 90% and a power of 80%, we would need to recruit a sample size of 313 patients, increased by 10% to account for missing values (*n* = 345).

Quantitative data are presented as medians with quartiles 1 and 3 due to the nonparametric distribution. Categorical variables are presented as numbers and percentages. The unpaired Wilcoxon rank sum test, Mann-Whitney U test and Kruskal-Wallis test were used to compare quantitative variables, and the chi-squared test or Fisher´s exact test to compare categorical variables. Sensitivity, specificity, positive and negative predictive values, positive and negative likelihood ratios and accuracy were calculated for the different predictive markers. Receiver operating characteristics (ROC) curves were constructed for each marker. DeLong’s test was used to compare differences in areas under the curves (AUC) between the curves and Youden index to determine the cutoff point. Decision curve analysis (DCA) was performed to evaluate the clinical utility of SeptiScore and comparator biomarkers (procalcitonin and C-reactive protein), estimating net benefit across a range of threshold probabilities and comparing performance against treat-all and treat-none strategies.

Correlation analysis was used to determine the relationship between the SeptiScore and other numeric variables using Pearson’s correlation coefficient (PCT levels were log-transformed for this analysis). Multivariable logistic regression analysis, using manual forward stepwise selection based on likelihood ratio, was performed with different biomarkers, clinical signs and physicians´ subjective likelihood of sepsis at ICU admission transformed as a dummy variable with low probability as the reference category. The likelihood ratio criterion and its overall validity using Nagelkerke´s R2 index were used to assess model fit and the proportion of variance in the dependent variable explained by the predictor variables. The Hosmer-Lemeshow test was also used to assess the overall goodness of fit of each model. Results are presented as odds ratio (OR) and 95% confidence interval (CI). A p value of < 0.05 was considered as statistically significant. SPSS 20.0 (IBM SPSS, Chicago, IL) and MedCalc 22.023 softwares were used for the statistical analyses.

## Results

### Study population

**Fig. 2:**
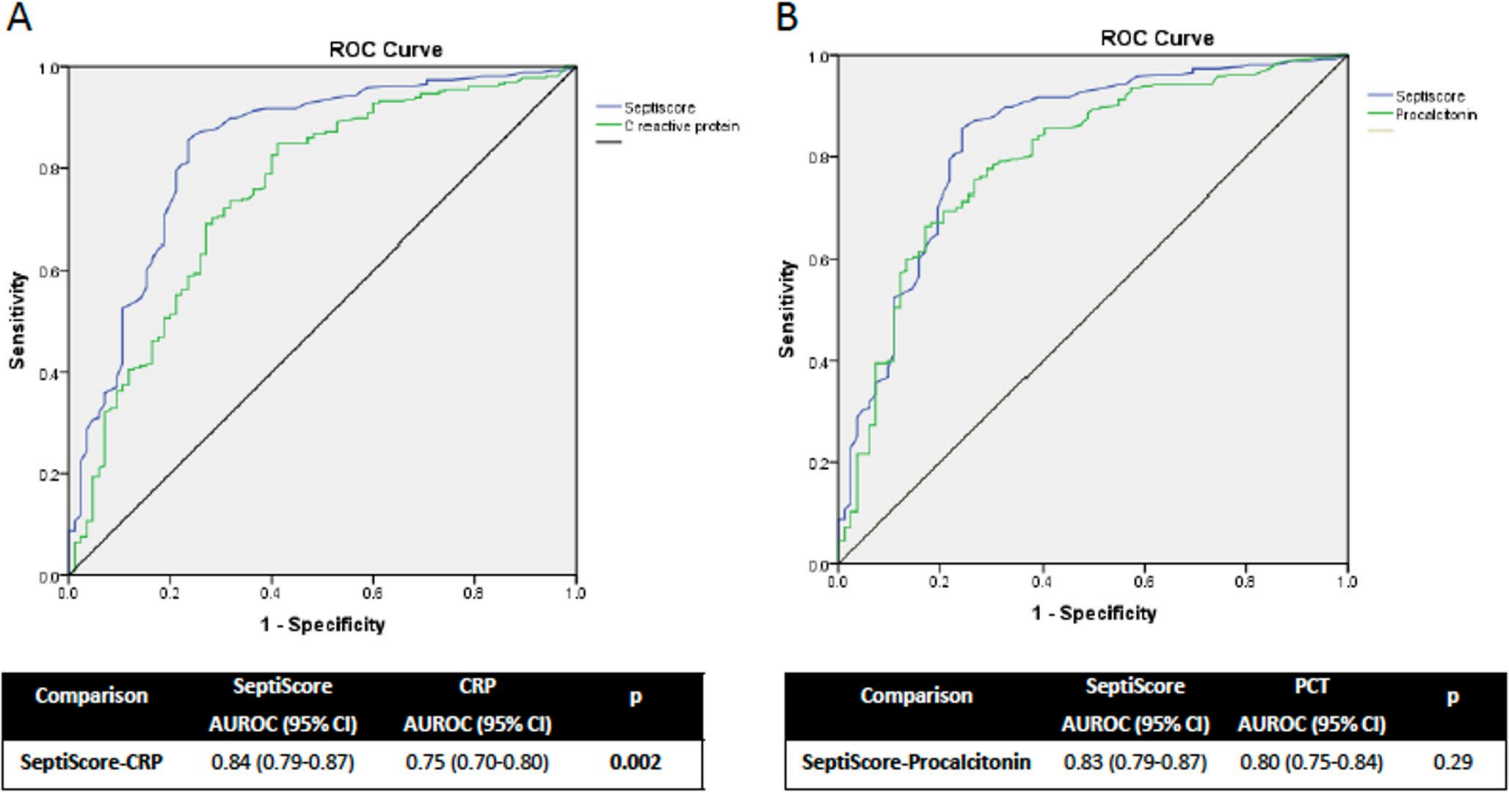
Comparison SeptiScore-CRP; 2B. Comparison SeptiScore-Procalcitonin

The study cohort included 368 patients but finally 353 patients were evaluable (S-Figure 2). The number of patients included in each participating unit is shown in S-Figure 3. The final diagnoses of the 86 patients admitted with suspicion of sepsis but finally diagnosed as non-infectious critical illness without underlying infection and the final diagnoses of the 18 patients withouth sepsis but Septiscore Band 4 are shown in S-Table 2 and S-Table 3 of the SDC, respectively. Patient characteristics are shown in Table 1. The baseline characteristics of the two groups (patients with sepsis vs. non-infectious critical illness) were comparable in terms of age, sex, APACHE II, and SOFA score. ICU and hospital mortality and length of stay were similar in both groups. Differences in laboratory data between patients with sepsis/non-infectious illness established at ICU discharge are depicted in S-Table 4.

**Table 1:** Analysis of demographics and clinical differences between patients with sepsis/non-infectious critical illness established at ICU discharge

Variable*		All patients (*n* = 353)	Sepsis (*n* = 267)	Non-infectious critical illness (*n* = 86)	*p*
Age		63 (54–71)	63 (54–71)	61 (52–70)	0.232
Gender	Male	219 (62.0%)	158 (59.2%)	61 (70.9%)	0.051
BMI		27.3 (24.2–32.0)	27.3 (24.2–31.8)	27.4 (24.2–33.1)	0.867
Comorbidities	Diabetes mellitus	117 (33.1%)	80 (30.0%)	37 (43.0%)	0.025
	Renal insufficiency	49 (13.9%)	35 (13.1%)	14 (16.3%)	0.460
	Immunosuppression	47 (13.3%)	34 (12.7%)	13 (15.1%)	0.572
	Neutropenia	8 (2.3%)	7 (2.6%)	1 (1.2%)	0.381
	Solid neoplasm	61 (17.3%)	53 (19.9%)	8 (9.3%)	0.024
	Hepatic cirrhosis	17 (4.8%)	13 (4.9%)	4 (4.7%)	0.599
	COPD	48 (13.6%)	31 (11.6%)	17 (19.8%)	0.055
	Cardiac insufficiency	23 (6.5%)	14 (5.2%)	9 (10.5%)	0.088
	Autoimmune diseases	18 (5.1%)	13 (4.9%)	5 (5.8%)	0.457
	SARS–CoV2 infection**	17 (4.8%)	15 (5.6%)	2 (2.3%)	0.172
	None of previous	124 (35.1%)	103 (38.6%)	21 (24.4%)	0.017
Type of ICU admission	Medical condition	280 (79.3%)	203 (76.0%)	77 (89.5%)	0.018
	Scheduled surgery	8 (2.3%)	6 (2.2%)	2 (2.3%)	
	Urgent surgery	65 (18.4%)	58 (21.7%)	7 (8.1%)	
Acquisition of illness	Community	267 (75.6%)	200 (74.9%)	67 (77.9%)	0.573
	Nosocomial	86 (24.4%)	67 (25.1%)	19 (22.1%)	
Heart rate		110 (90–120)	110 (96–120)	100 (80–118)	< 0.001
Respiratory rate		22 (18–28)	22 (18–28)	20 (18–30)	0.712
Mean arterial pressure		64 (54–77)	62 (53–73)	73 (60–83)	< 0.001
APACHE II score		18 (14–24)	19 (14–24)	17 (12–25)	0.255
SOFA score		7 (5–9)	7 (5–9)	6 (4–9)	0.139
Septiscore		8.4 (6.5–10.1)	9.2 (7.6–10.5)	5.8 (4.6–6.8)	< 0.001
Band of Septiscore	BAND 1 (0–4.9.9)	35 (9.9%)	9 (3.4%)	26 (30.2%)	< 0.001
	BAND 2 (5.0–6.1.0.1)	40 (11.3%)	13 (4.9%)	27 (31.4%)	
	BAND 3 (6.2–7.3)	48 (13.6%)	33 (12.4%)	15 (17.4%)	
	BAND 4 (7.4–15)	230 (65.2%)	212 (79.4%)	18 (20.9%)	
Shock	No shock	131 (37.1%)	85 (31.8%)	46 (53.5%)	< 0.001
	Shock	222 (62.9%)	182 (68.2%)	40 (46.5%)	
Physicians´ subjective likelihood of sepsis at ICU admission	< 30%	40 (11.3%)	9 (3.4%)	31 (36.0%)	< 0.001
	30–70%	97 (27.5%)	50 (18.7%)	47 (54.7%)	
	> 70%	216 (61.2%)	208 (77.9%)	8 (9.3%)	
Blood culture at admission	Positive	105 (29.7%)	101 (37.8%)	0 ***	< 0.001
Suspected Source	Abdominal	116 (32.9%)	94 (35.2%)	22 (25.6%)	0.098
	Bacteremia	19 (5.4%)	18 (6.7%)	1 (1.2%)	0.046
	Respiratory	110 (31.2%)	76 (28.5%)	34 (39.5%)	0.054
	Catheter related	5 (1.4%)	4 (1.5%)	1 (1.2%)	0.646
	Urological	38 (10.8%)	32 (12.0%)	6 (7.0%)	0.192
	Soft tissue	13 (3.7%)	11 (4.1%)	2 (2.3%)	0.348
	CNS	19 (5.4%)	13 (4.9%)	6 (7.0%)	0.305
	Other foci****	33 (9.3%)	19 (7.1%)	14 (16.3%)	0.013
RRT first 72 h	Yes	77 (21.8%)	51 (19.1%)	26 (30.2%)	0.030
IMV first 72 h	Yes	186 (52.7%)	143 (53.6%)	43 (50.0%)	0.565
Vasopressors first 72 h	Yes	228 (64.6%)	190 (71.2%)	38 (44.2%)	< 0.001
ICU stays (days)		5 (2–12)	5 (2–14)	4 (2–9)	0.143
Hospital stays (days)		16 (8–35)	17 (8–36)	14 (8–27)	0.124
ICU death	Yes	76 (21.5%)	59 (22.1%)	17 (19.8%)	0.648
Hospital death	Yes	93 (26.4%)	71 (26.7%)	22 (25.6%)	0.839

*Result expressed with median (p25-p75) or number (percentage) as appropriate

**Symptoms in previous 30 days

*** 4 coagulase-negative Staphylococci, considered contaminant by the researches

****Other foci: Cardiovascular; Gynaecological; Surgical wound; Ear, nose and throat; Bones and Unknown source

APACHE II: Acute Physiology and Chronic Health disease Classification System II; BMI: Body Mass Index; CNS: Central nervous system; COPD: Chronic obstructive pulmonary disease; ICU: Intensive Care Unit; IMV: Invasive mechanical ventilation; RRT: Renal replacement therapy; SOFA: Sequential Organ Failure Assessment Score

CRP: C- reactive protein; PCT: Procalcitonin. ROC curve: Receiver operating characteristics curve; AUROC: Area under receiver operating characteristics curve

Bacteremia was detected in 105 patients on admission to the ICU, and the source was documented in 50.6% of patients with sepsis. Microbiological results (blood cultures and cultures from the different foci) are shown in S-Table 5 and S-Table 6. Of the 267 patients adjudicated as having sepsis, 176 (65.9%) had concordant microbiological documentation of the infection. For completeness, these 176 microbiologically documented cases represent 49.9% of the whole evaluable cohort (176/353).

Physicians’ high subjective probability of sepsis at ICU admission was a good predictor of sepsis, with a sensitivity of 77.9%, specificity of 90.7%, positive predictive value of 96.3%, negative predictive value of 56.9% and accuracy of 81%. A definitive diagnosis of sepsis at ICU discharge in the low probability group (< 30%) was 22.5%, and 51.5% in the intermediate probability group (30–70%).

### SeptiCyte^®^RAPID performance: diagnostic value

SeptiScores were significantly higher in patients with sepsis compared to those with non-infectious critical illness (Fig. 1; Table 1). The scores of patients from the two groups were distributed among the four interpretation bands as shown in Table 1.

The optimal SeptiScore cut-off for the diagnosis of sepsis, determined by maximizing sensitivity and specificity (Youden index), was 6.9. S-Table 7 presents the diagnostic performance parameters (sensitivity, specificity, PPV, NPV, likelihood ratios, and accuracy) corresponding specifically to this cut-off.

The clinical performance of the 4 bands for the diagnosis of sepsis is shown in Table 2 and S-Figure 4. Patients with a higher SeptiScore were more likely to have sepsis. When analyzed according to bands (1 to 4, as defined by the manufacturer): the PPV for sepsis increased from 25.7% in Band 1 to 92.2% in Band 4 (Table 2). For the diagnosis of sepsis, Band 4 had a sensitivity of 79.4%, a PPV of 92.2% and NPV of 55.3%, with a positive likelihood ratio of 3.79, a negative likelihood ratio of 0.26, and an accuracy of 79.3%. The S-Figure 5 depitcs the Fagan nomongram of Band 4 showing the probability of this positive result depending of the pre-test probability. In addition, among patients with a low physician-estimated probability of sepsis at ICU admission, 18 individuals presented SeptiScores within Bands 1–2, and none of them were ultimately adjudicated as sepsis. This subgroup therefore showed a PPV of 100% for the absence of sepsis.

**Table 2:** Clinical performance of the 4 bands for the diagnosis of sepsis

Septiscore for sepsis	Sensitivity(CI95%)	Specificity(CI95%)	PPV(CI95%)	NPV(CI95%)	LR +(CI95%)	LR -(CI95%)	AC(CI95%)
**BAND 1 (0–4.9.9)**	3.4%(1.6%−6.3%)	69.8%(58.9%−7.2%)	25.7%(14.4%−41.5%)	18.9%(16.8%−21.2%)	0.11(0.05–0.23)	1.39(1.20–1.60)	19.5%(15.6%−24.1%)
**BAND 2 (5.0–6.1.0.1)**	4.9%(2.6%−8.2%)	68.6%(57.7%−78.2%)	32.5%(20.6%−47.1%)	18.8%(16.8%−21.2%)	0.16(0.08–0.29)	1.39(1.20–1.60)	20.4%(16.4%−25%)
**BAND 3 (6.2–7.3)**	12.4%(8.7%−16.9%)	82.6%(72.9%−89.9%)	68.8%(55.6%−79.4%)	23.3%(21.5%−25.3%)	0.71(0.42–1.24)	1.06(0.95–1.18)	29.5%(24.8%−34.5%)
**BAND 4 (7.4–15)**	79.4%(74%−84.1%)	79.1%(69%−87.1%)	92.2%(88.6%−94.7%)	55.3%(48.9%−61.6%)	3.79(2.50–5.75)	0.26(0.20–0.34)	79.3%(74.7%−83.4%)

AC: Accuracy; LR: Likelihood ratio; NPV: Negative predictive value; PPV: Positive predictive value

In patients with the final diagnosis of sepsis, the proportion of Band 4 in bacteremic vs. non-bacteremic patients and in patients with microbiologically documented sepsis vs. non-microbiologically documented episodes of sepsis was not statistically different, 9.1% difference [CI 95% −0.9% to 18.2%]; *p* = 0.073 and 5.5% difference [CI 95% −4.5% to 16.5%]; *p* = 0.293, respectively. Patients with diabetes mellitus and solid neoplasm were not equally distributed in patients with sepsis and non-infectious critical illness (Table 1). Levels of septiscore were not different in patients with or without neoplasm, but septiscore was lower in diabetic patients (8.8 [6.8–10.5] vs.7.7 [5.8–9.5]; *p* < 0.001)

In addition, in these patients with the final diagnosis of sepsis, Septiscore values were not statistically different in patients with sepsis and septic shock, as well as between bacteremic and non-bacteremic patients (S-Figs. 6 and 7).

### Comparison of SeptiCyte^®^ RAPID performance with PCT and CRP

Statistical comparison of ROC curves (Fig. 2A and B, and S-Figure 8) showed that SeptiScore [AUC 0.84 (95%CI 0.79–0.87)] was significantly better than CRP [AUC 0.75 (95%CI 0.70–0.80)] at identifying the presence of sepsis in patients on admission to the ICU, (DeLong’s test *p* = 0.003), although, the AUCs for SeptiScore and PCT [0.80 (95% CI 0.75–0.84)] were not statistically different (DeLong’s test, *p* = 0.29).

Diagnostic performance of Septiscore alone was not improved combining SeptiScore cut-off-point of maximum sensitivity and specificity with the CRP or PCT cut-off points (S-Table 7 and S-Table 8).

Decision curve analysis showed that SeptiScore provided a higher or comparable net clinical benefit than PCT within low-to-intermediate decision thresholds of approximately 20–50%, which correspond to the clinical scenario with the greatest diagnostic uncertainty at ICU admission. At higher thresholds (≥ 60–70%), the net benefit of all biomarkers declined and approached the “treat none” strategy, suggesting that in these cases management is largely driven by clinical judgment alone. The combined model, integrating SeptiScore, PCT, and clinical variables, achieved the highest net benefit across most threshold ranges, supporting its potential role as the preferred decision strategy.(S-Figure 9).

## Multivariable analysis for prediction of sepsis

Multivariable logistic regression analysis (Table 3) including the physicians´ subjective likelihood of sepsis, CRP, log procalcitonin, leukocytes, neutrophils proportion, lactate, and SeptiScore showed that the independent variables associated with sepsis were physicians´ subjective likelihood of sepsis [intermediate probabilty OR 4.55 (95% CI 1.61–12.83), *p* = 0.004 and high probabilty OR 72.04 (95% CI 20.85–248.93.85.93), *p* < 0.001], SeptiScore [OR 1.65 (95% CI 1.32–2.07), *p* < 0.001] and log procalcitonin [OR 1.74 (95% CI 1.08–2.79), *p* = 0.022]. In patients with clinically low probability of sepsis (< 30%), the multivariate analysis with introducing Septiscore, log procalcitonin, CRP, leukocytes, neutrophils, lactate, SOFA and APACHE II scores at ICU admission revealed that only SeptiScore could independently be associated with the diagnosis of sepsis [OR 1.98 (95% CI 1.23–3.17), *p* < 0.005].

**Table 3:** Multivariate analysis for sepsis

	OR (CI 95%)	*p*
Physicians´ subjective likelihood of sepsis	8.02 (4.50–14.28.50.28)	< 0.001
Septiscore	1.64 (1.35–1.99)	< 0.001
Log procalcitonin	1.68 (1.09–2.59)	0.020
C reactive protein	1.00 (0.99–1.00.99.00)	0.541
Lactate	0.92 (0.80–1.07)	0.273
Neutrophils proportion	7.26 (0.43–122.51.43.51)	0.169
Leucocytes	0.98 (0.95–1.01)	0.116

CI: Confidence interval; OR: Odds ratio

Regarding clinical outcomes, SeptiScore did not demonstrate a significant association with disease severity or mortality. Systematic ROC curve analyses evaluating SeptiScore as a predictor for both ICU and in-hospital mortality showed poor performance, and these data are presented in S-Figs. 10 and 11.

## Discussion

The results of this prospective study confirm the validity of SeptiCyte^®^ RAPID for differentiating sepsis from non-infectious critical illness in critically ill adult patients meeting Sepsis-3 criteria on admission to the ICU. In this study, which included medical-surgical patients, performance of the SeptiCyte^®^ RAPID assay for the diagnosis of sepsis was not statistically different from the performance of PCT, but was significantly better than CRP. In the multivariable analysis, SeptiScore added to the physicians´ subjective likelihood of sepsis and improved the accuracy of sepsis diagnosis.

In previous studies, both SeptiCyte^®^ LAB [10–12] and SeptiCyte^®^ RAPID [21] demonstrated the ability to differentiate sepsis from non-infectious systemic inflammation, generally reporting AUCs within the 0.7–0.9 range depending on cohort design and assay format. These studies established the biological rationale for a host-response transcriptomic approach but were often limited by their retrospective designs and the use of Sepsis-2 criteria. By contrast, our work assesses SeptiCyte^®^ RAPID prospectively in a multicenter ICU cohort using Sepsis-3 definitions, thereby providing new insights about this assay applicability in the current clinical practice.

Both infectious and non-infectious critical illnesses activate similar immune pathways leading to the expression of the same proinflammatory genes and cytokines. However, several genes have been identified as specific to pathogen-associated molecular patterns [22]. A recent study identified nineteen differentially expressed mRNA biomarkers able to distinguish sepsis from non-infectious critical illness [23]. Two of these specific genes (PLA2G7 and PLAC8) make up the SeptiCyte^®^ RAPID transcriptomic assay, which measures the expression levels of these two genes and returns the results in approximately 60 min.

In this prospective study, the diagnostic performance of SeptiCyte^®^ RAPID [AUC = 0.84 (95% CI: 0.79–0.89)] was similar to that reported in previous studies for SeptiCyte^®^ LAB [10, 11]. Thus, in one study combining the results of three prospective, observational studies, SeptiCyte^®^ LAB had an AUC of 0.82–0.89 for differentiating sepsis from non-infectious critical illness [11]. In a retrospective study of critically ill patients with low mortality (ICU mortality 5.5%), the AUC was 0.80 (95% CI 0.68–0.93) when only blood culture-positive patients were analyzed, and 0.79 (95% CI 0.68–0.90) when blood-culture negative sepsis episodes were also included [24]. However, the discriminative power for infection was lower for SeptiCyte^®^ LAB 0.731 (95% CI, 0.677–0.786) in previously hospitalized patients admitted to the ICU with acute respiratory insufficiency [12]. The performance of SeptiCyte^®^ RAPID has been assessed by reanalyzing samples obtained from two retrospective cohorts of adult patients with SIRS and a prospective cohort of 63 patients. Patients were enrolled on the first day of ICU admission and the performance was significantly different between the retrospective and prospective cohorts [21]. More recently, the utility of SeptiCyte^®^ RAPID has been investigated in cohort of 61 patients undergoing scheduled abdominal surgery. The AUC of the sample obtained ± 3 h around the time of blood culture extraction for discriminating between sepsis from inflammation was modest: 0.71 (CI 95% 0.58–0.85) [25].

For patients with a low probability of sepsis, the latest version of the Surviving Sepsis Campaign guidelines [26] recommends deferring initiation of antibiotics and assessing other likely etiologies. A SeptiScore Band of 1or 2 for patients with a physicians´ low subjective likelihood of sepsis had a specificity and PPV of 100% to support not prescribing antibiotics, which is favorable for antimicrobial stewardship programs. Conversely, a SeptiScore Band 4 would provide additional evidence of an infectious etiology with a 96.5% specificity before positive microbiology results are available.

We evaluated SeptiCyte^®^ RAPID performance in comparison to CRP and PCT, the biomarkers most commonly used for the diagnosis of sepsis in clinical practice [27, 28]. In our study, the AUC of PCT for the diagnosis of sepsis was slightly lower than the AUC obtained in a meta-analysis that combined data from 30 studies to assess the accuracy of PCT as a diagnostic marker of sepsis [AUC 0.85 (95% CI 0.81–0.88)] [29]. In a recent study evaluating critically ill patients on antibiotics, the performances of SeptiCyte^®^LAB and PCT were similar [26]. It should be note that the cut-off values for distinguishing sepsis patients from those with non-infectious critical illness vary widely among studies. We also found that the ability of SeptiScore to identify sepsis was significantly better than the accuracy of CRP. Multivariable analysis showed that the combination of SeptiScore with PCT and the physicians´ subjective likelihood of sepsis significantly improved the overall diagnostic performance for identifying sepsis. Moreover, SeptiScore could be better than procalcitonin and CRP to detect sepsis in patients with low probability of sepsis at ICU admission.

In our study, higher SeptiScores were not associated with greater disease severity on ICU admission or mortality. Since SeptiCyte^®^ RAPID scores are based on gene activation, the SeptiScore value depends on the presence of an invasive infection and is not affected by the disease severity. In contrast, two recent studies [30, 31] have described the ability of SeptiCyte^®^ RAPID to stratify COVID-19 cases according to clinical severity by predicting the need for ICU admission. Very likely, the inflammatory response associated with COVID-19 patients is different from the response in bacterial sepsis [32, 33].

Beyond conventional diagnostic accuracy metrics, decision curve analysis provides a clinical utility framework by incorporating the consequences of false-positive and false-negative classifications across a spectrum of pre-treatment probabilities. In this context, SeptiScore consistently yielded higher net benefit in low-to-moderate probability ranges, which corresponds to the early triage phase when the decision to initiate antibiotics is most uncertain. At higher probabilities of sepsis, biomarker-guided strategies offered little additional net benefit over clinical judgment alone, indicating that treatment decisions are primarily determined by the overall clinical picture in these patients.​.

Moreover, the combined model integrating SeptiScore, PCT, and clinical variables provided the greatest net benefit over most of the evaluated threshold range, underscoring the value of combining host-response transcriptomics with established biomarkers and bedside clinical assessment to support decision-making in the critical care setting.

We acknowledge several limitations of this study. First, only adult patients admitted to the ICU were included, so that generalizability to other patient cohorts could not be determined. Second, as an observational study, the patient population was quite heterogenous in terms of pre-existing conditions, comorbidities and concomitant treatments, some of which may have influenced the SeptiCyte^®^ RAPID results. Third, administration of antibiotics before ICU admiission was not recorded in the present study. Therefore, the impact of the use of antibiotics on SeptiCyte^®^ RAPID results cannot be assessed. Finally, the difficulty of evaluating the performance of new diagnostic tests in sepsis patients, where there is no gold-standard, is an inherent limitation of this type of study.

Several strengths of our study should also be highlighted. First, this is the first study to evaluate the performance of SeptiCyte^®^ RAPID in a prospective cohort, for the diagnosis of sepsis. Second, this is a prospective, multicenter study, with a relatively large number of patients enrolled, which reflects the real-life of patients with sepsis. Third, we included medical-surgical ICU patients who fulfilled the criteria for sepsis or septic shock according to the new Sepsis-3 criteria [16].

To sum up, this is the largest study that has prospectively evaluate the new SeptiCyte^®^ RAPID test confirming that this new rapid test successfully differentiates sepsis from non-infectious critical illness in patients admitted to the ICU under the Sepsis-3 criteria. Used in conjunction with PCT and clinical judgment, SeptiScore can increase physician confidence in therapeutic decision-making. SeptiCyte^®^ RAPID may thus provide utility to support not prescribing antibiotics in patients with bands 1 or 2 and low probability of sepsis, which is favorable for, antimicrobial stewardship programs. Well-designed clinical studies in different patient populations are needed to confirm the ability of SeptiCyte^®^ RAPID to improve clinical outcomes.

## Supplementary Information

Below is the link to the electronic supplementary material.

Supplementary Material 1 (DOCX 4.89 MB)

## Data Availability

The datasets used and/or analysed during the current study are available from the corresponding author on reasonable request.
