# Supplementary material for: Diagnostic accuracy of SeptiCyte® RAPID to discriminate sepsis from non-infectious critical illness in patients meeting sepsis criteria according to sepsis-3 definition at ICU admission
Source: Eur J Clin Microbiol Infect Dis. 2026 Feb 23;45(6):1605–15. doi: 10.1007/s10096-026-05431-6 (PMC13319364; doi:10.1007/s10096-026-05431-6)

SUPPLEMENTAL DIGITAL CONTENT

Contents

[STUDY PROTOCOL 4](#_Toc218331041)

[Primary objective 4](#_Toc218331042)

[Secondary objectives 4](#_Toc218331043)

[Appendix 1 7](#_Toc218331044)

[METHODS 8](#_Toc218331045)

[RESULTS 9](#_Toc218331046)

[Supplemental Table 1. STARD list. 9](#_Toc218331047)

[Supplemental Table 2. Final diagnoses in patients with ~~sterile inflammation~~ non-infectious critical illness. 12](#_Toc218331048)

[Supplemental Table 3. Final diagnoses in patients without sepsis but Septiscore Band 4 12](#_Toc218331049)

[Supplemental Table 4. Analysis of laboratory data differences between patients with sepsis/~~sterile inflammation~~ non-infectious critical illness established at ICU discharge. 13](#_Toc218331050)

[Supplemental Table 5. Micro-organisms isolated in blood cultures. 14](#_Toc218331051)

[Supplemental Table 6. Micro-organisms isolated in source of infection. 15](#_Toc218331052)

[Supplemental Table 7. Cut-off point of maximum sensitivity and specificity of Septiscore. 16](#_Toc218331053)

[Supplemental Table 8. Cut-off point of maximum sensitivity and specificity of Septiscore combined with Procalcitonin or C-reactive protein cut-off points (Youden test) 17](#_Toc218331054)

[Supplemental Table 9. Correlation between Septiscore and other markers and scores 17](#_Toc218331055)

[Supplemental Table 10. Demographics, clinical and analytical characteristics of patients with sepsis and Band 1 and non-infectious critical illness and Band 4 established at ICU discharge. 18](#_Toc218331056)

[Supplemental Figure 1. Sepsis Probability Bands (according to the manufacturer). 20](#_Toc218331057)

[Supplemental Figure 2. Flowchart of the patients included in this study. 20](#_Toc218331058)

[Supplemental Figure 3. Number of valid cases in every hospital. 21](#_Toc218331059)

[Supplemental Figure 4. Clinical performance of the 4 bands for the diagnosis of sepsis. 21](#_Toc218331060)

[Supplemental Figure 5. Fagan nomogram for Septiscore BAND 4. 22](#_Toc218331061)

[Supplemental Figure 6. Comparation of Septiscore values between definitely septic patients with sepsis or septic shock. Boxplots and violin plots. 23](#_Toc218331062)

[Supplemental Figure 7. Comparation of Septiscore values between definitely septic patients bacteremic and not bacteremic. Boxplots and violin plots. 23](#_Toc218331063)

[Supplemental Figure 8. Curves for SeptiScore, PCT, CRP and results of the pairwise AUROC comparisons. 25](#_Toc218331064)

[Supplemental Figure 9. Comparation of models to predict Sepsis. 26](#_Toc218331065)

[Supplemental Figure 10. Performance of Septicyte and PCT as predictors of in-hospital mortality 26](#_Toc218331066)

[Supplemental Figure 11. Performance of Septicyte and PCT as predictors of ICU mortality 27](#_Toc218331067)

[Supplemental Figure 12. Correlation between Septiscore and other markers and scores 27](#_Toc218331068)

STUDY PROTOCOL

Diagnostic accuracy of SeptiCyte for detection of underlying infection in critically ill patients with Sepsis-3 criteria on admission to the ICU.

**Principal investigator**. Dr. José Garnacho Montero. (University Hospital Virgen del Rocío).

Primary objective

To determine the diagnostic accuracy (Sensitivity, specificity, positive and negative predictive values, and positive and negative likelihood ratios) of the SeptiCyte® RAPID (Septiscore) in patients admitted to the ICU with sepsis criteria according to Sepsis-3 definition.

Secondary objectives

To know if SeptiCyte® RAPID (Septiscore) values are different in sepsis and septic shock.

To determine if SeptiCyte® RAPID (Septiscore) values can identify patients with bacteremia.

To explore if SeptiCyte® RAPID (Septiscore) values are different in patients with community-acquired or hospital-acquired sepsis/inflammation.

Compare the diagnostic value of SeptiCyte® RAPID (Septiscore) with C-reactive protein and procalcitonin values.

Determine the characteristics of the population for very low probability of sepsis on ICU admission (SeptiCyte® RAPID < 5 and PCT < 0.25 ng/mL) in whom antibiotic treatment might not be initiated.

Methods

Participant Units:

Andalucía Region: Seven ICUs : ICU of H Virgen Macarena (Seville), H Virgen del Rocío (Seville), H de Jerez (Jerez, Cádiz), H San Juan de Dios (Seville), H Reina Sofía (Córdoba), H Clínico de Málaga (Málaga), H Universitario Regional de Málaga (Málaga).

Inclusión criteria:

Patients with suspicion of sepsis according to Sepsis-3 definition at ICU admission.

Exclusion criteria:

Patients < 18 years of age.

Pregnant woman

Signs and symptoms suggestive of sepsis had been present for more than 48 hours.

Variables

Demographic characteristics.

Underlying diseases (see definitions in Appendix 1)

Severity of illness (worst APACHE II in the first 24 h).

Organ dysfunction on admission (worst SOFA in the first 24 h).

Type of ICU admission: Medical condition, Scheduled surgery or Urgent surgery

Type of suspected infection (community-acquired or hospital-acquired)

Clinical Focus of suspected sepsis.

Subjective likelihood of sepsis at ICU admission by the attending physician (low < 30%, intermediate 30-70%, high >70%), according to the clinical and analytical data. Focus of sepsis.

Presence of bacteremia.

Isolated microorganisms in blood and/or focus.

Blood count.

Creatinine, urea.

Lactate.

SeptiCyte® RAPID (Septiscore)

C-reactive protein and procalcitonin.

Use of vasopressors.

Need of invasive mechanical ventilation in the first 72 hours.

Need of renal replacement therapy in the first 72 hours.

Follow-up until death or hospital discharge.

Study protocol

Blood samples will be drawn on the admission day (as soon as the diagnosis of sepsis is done) for: blood culture, blood count, general blood biochemistry including CRP and PCT levels. SeptiCyte® RAPID. Blood samples for the SeptiCyte® RAPID assay will be collected in PAXgene® Blood RNA tubes, and cooled to 2-8º C.

Cultures of the suspected focus of infection will be taken as clinically indicated.

Imaging will be done by the physician in charge of the patient as clinically indicated.

Infection diagnosis:

It will be performed in each case by 2 investigators of this project belonging to each hospital considering the clinical, analytical, radiological, and microbiological data and blinded to the SeptiCyte® RAPID results. All patients with positive microbiology (blood and/or focus) will be categorized as Sepsis. As exceptions, skin commensals as coagulase-negative Staphylococci, Corynebacterium spp., and Bacillus spp. isolated in a single blood cultures or in focus (urine, respiratory sample) will be considered as contaminants.

In patients with negative microbiology, the diagnosis of sepsis will be established by two local investigators based on the presence of clinical, radiological, and analytical features strongly suggestive of infection. Furthermore, the Steering Committee (MLCB, AEG, JLGG, and JGM) will review all cases and contacted local investigators in case of doubts.

Management of the patients will be done in accordance with local protocols.

Ethical aspects

Study approval will be requested from the institutional review board (IRB). Since it is very important to obtain these data on admission and without delay, it will be performed as part of the routine analysis and for SeptiCyte® RAPID 1 mL of whole blood is required. Subsequently, informed consent will be requested from the patient or family member if the patient is unable to give it and will not be included in the study if such consent is denied. This must be approved by the IRB of the participating hospitals.

Statistical analysis

Sample size: based on a previous study (Garnacho-Montero J, et al. Prognostic and diagnostic value of eosinopenia, C-reactive protein, procalcitonin, and circulating cell-free DNA in critically ill patients admitted with suspicion of sepsis. Crit Care. 2014;18:R116) 75% of the patients admitted to the ICU with the clinical suspicion of sepsis will have a confirmed sepsis. Assuming that the clinical assessments have a sensitivity and a specificity of 80%, and for the SeptiCyte® RAPID test a sensitivity and a specificity of 95%, we estimated that with a confidence level of 90% and a power of 80%, we will need to recruit a sample size of 313 patients, increased by 10% to account for missing values (n=345).

Analysis: Data will be expressed as means or medians with measures of dispersion according to the normality of distribution. Quantitative analyses will be performed using ANOVA or t-Student if the distributions are normal and Kruskal-Wallis or Mann-Whitney U in case of asymmetric distributions. Chi-square or Fisher's exact test will be used for qualitative analysis. Logistic regression analysis will be performed using various indicators to determine independent predictive capability.

Sensitivity, specificity, positive and negative predictive values, and positive and negative likelihood ratios will be performed. ROC curves will be constructed and the difference between them will be calculated using the DeLong test. All tests will be bilateral and a significance p<0.05 will be used. SPSS 20.0 (IBM SPSS, Chicago, IL) and MedCalc 22.023 softwares will be used for the statistical analyses.

Appendix 1

Diabetes mellitus: pre-existing, physician-diagnosed disorder of glucose metabolism documented before ICU admission requiring medical treatment.

Renal insufficiency: Chronic renal failure requiring dialysis.

Immunosuppression: Patients receiving immunosuppressive therapy (chemotherapy, long-term steroids, radiation) or with advanced diseases like AIDS, leukemia, lymphoma, or multiple myeloma.

Neutropenia: Neutrophil Count < 500 cells/µL.

Solid neoplasm: Presence of a diagnosed cancer that has not been cured or is still active at admission (e.g., ongoing treatment, known residual disease). This excludes past malignancies in remission.

Hepatic cirrhosis: clinically diagnosed liver cirrhosis, severe hepatitis.

COPD: Severe chronic obstructive lung disease, chronic restrictive disease with severe exercise limitation or chronic hypoxia requiring home oxygen therapy.

Cardiac insufficiency: Congestive heart failure (Class III or IV) or severe cardiomyopathy.

Autoimmune diseases. Any Autoimmune disease requiring immunosuppressive therapy including corticosteroids.

SARS–CoV2 infection: COVID-19 in the previous 30 days.

METHODS

SeptiCyte® RAPID assay

As soon as possible after admission to the ICU, a blood sample was obtained from the patient for determination of PCT and CRP levels and for the SeptiCyte® RAPID assay. Blood samples for the SeptiCyte® RAPID assay were collected in PAXgene® Blood RNA tubes, and cooled to 2-8º C, according to the manufacturer´s instructions. These PAXgene® Blood RNA samples were gently inverted 10 times to ensure homogeneity of tube contents. The Idylla™ platform (Biocartis NV, Mechelen, Belgium) was available in 4 of the participants Units: Hospital Universitario Virgen Macarena (Sevilla), Hospital Universitario de Jerez de la Frontera (Cádiz), Hospital Universitario Virgen de la Victoria (Málaga), and Hospital Universitario Reina Sofía (Córdoba). Samples obtained in Hospital San Juan de Dios del Aljarafe (Sevilla) and Hospital Universitario Virgen del Rocío (Sevilla) were sent for processing in less than 4 days to the Hospital Universitario Virgen Macarena (Sevilla) and samples from Hospital Universitario Regional de Málaga (Málaga) were sent to the Hospital Universitario Virgen de la Victoria (Málaga). Temperature conditions were maintained during transport of samples between hospitals.

Samples were processed in less than five days. A 0.9 mL aliquot was pipetted into the SeptiCyte® RAPID cartridge, and processed according to SeptiCyte® RAPID instructions for use. Analysis to produce the SeptiCyte® RAPID scores (SeptiScore) was performed automatically on the Idylla™ platform, which is capable of fully automated nucleic acid testing, including extraction, amplification, and detection, all in approximately one hour.

The performance of the SeptiCyte® RAPID assay was compared with the clinical diagnosis made by the research team. CRP and PCT levels were measured in the clinical laboratory in each participating hospital. CRP was determined by immunoturbidimetric assay on a Modular P Chemistry analyzer (Roche Diagnostics, GmbH, Mannheim, Germany). The reference limit for this method is less than 0.5 mg/dL. PCT was measured by immunofluorescence assay using the BRAHMS PCT kit (Roche, Zurich, Switzerland) following the manufacturer’s protocols. The lower detection limit for this method is 0.05 ng/mL.

Missing values were treated by pairwise deletion of cases.

RESULTS

*Comparison of SeptiCyte® RAPID performance with PCT in patients with bacteremia.*

When the performance of the SeptiCyte® RAPID was compared with PCT in patients with positive blood cultures (n=106), no significant differences were observed between the two in differentiating between sepsis and sterile inflammation. non-infectious critical illness.

*Correlation between SeptiScore and biomarkers or clinical scores*

Statistically significant correlations were shown between SeptiScore and procalcitonin (log-transformed) and CRP (p<0.001) but not with the severity scoring systems (APACHE II and SOFA), age or BMI, as shown in S-Table 8 and S-Figure 7.

Descriptions of patients with Band 1 finally diagnosed of sepsis and with Band 4 and sterile inflammation non-infectious critical illness established at ICU discharge

Demographics, clinical and analytical characteristics of patients with sepsis and Band 1 and sterile inflammation non-infectious critical illness and Band 4 established at ICU discharge are shown in S-Table 9.

Supplemental Table 1. STARD list.

|  | **Section & Topic** | **No** | **Item** | **Reported on page #** |
| --- | --- | --- | --- | --- |
|  |  |  |  |  |
|  | **TITLE OR ABSTRACT** |  |  |  |
|  |  | **1** | Identification as a study of diagnostic accuracy using at least one measure of accuracy  (such as sensitivity, specificity, predictive values, or AUC) | Page 1 Tittle and abstract |
|  | **ABSTRACT** |  |  |  |
|  |  | **2** | Structured summary of study design, methods, results, and conclusions  (for specific guidance, see STARD for Abstracts) | Page 1-2 |
|  | **INTRODUCTION** |  |  |  |
|  |  | **3** | Scientific and clinical background, including the intended use and clinical role of the index test | Page 2-5, paragraphs 1–3 |
|  |  | **4** | Study objectives and hypotheses | Page 3-4 |
|  | **METHODS** |  |  |  |
|  | *Study design* | **5** | Whether data collection was planned before the index test and reference standard  were performed (prospective study) or after (retrospective study) | Page 4 “multicenter prospective cohort study” |
|  | *Participants* | **6** | Eligibility criteria | Page 4-5 Methods: inclusion and exclusion criteria |
|  |  | **7** | On what basis potentially eligible participants were identified  (such as symptoms, results from previous tests, inclusion in registry) | Page 4-5 “Patients admitted with diagnosis of sepsis according to Sepsis-3” |
|  |  | **8** | Where and when potentially eligible participants were identified (setting, location and dates) | Page 4-5 “seven ICUs in Andalusia, March–Dec 2022” |
|  |  | **9** | Whether participants formed a consecutive, random or convenience series | Page 4-5 |
|  | *Test methods* | **10a** | Index test, in sufficient detail to allow replication | Page 4-5 Methods: full description of SeptiCyte® RAPID and Sepsis-3 reference |
|  |  | **10b** | Reference standard, in sufficient detail to allow replication | Page 4-5 Methods: full description of SeptiCyte® RAPID and Sepsis-3 reference |
|  |  | **11** | Rationale for choosing the reference standard (if alternatives exist) | Page 4-5, clinical adjudication by investigators blinded to SeptiScore |
|  |  | **12a** | Definition of and rationale for test positivity cut-offs or result categories  of the index test, distinguishing pre-specified from exploratory | Page 4-5; S-Methods; SDC, SeptiCyte® RAPID procedure |
|  |  | **12b** | Definition of and rationale for test positivity cut-offs or result categories  of the reference standard, distinguishing pre-specified from exploratory | Page 4-5, clinical adjudication by investigators blinded to SeptiScore |
|  |  | **13a** | Whether clinical information and reference standard results were available  to the performers/readers of the index test | Page 4-5, clinicians and adjudicators blinded to SeptiScore |
|  |  | **13b** | Whether clinical information and index test results were available  to the assessors of the reference standard | Page 4-5, clinicians and adjudicators blinded to SeptiScore |
|  | *Analysis* | **14** | Methods for estimating or comparing measures of diagnostic accuracy | Page 6. full statistical analysis, ROC curves, multivariable logistic regression |
|  |  | **15** | How indeterminate index test or reference standard results were handled | Page 6. full statistical analysis, ROC curves, multivariable logistic regression |
|  |  | **16** | How missing data on the index test and reference standard were handled | Page 6. full statistical analysis, ROC curves, multivariable logistic regression |
|  |  | **17** | Any analyses of variability in diagnostic accuracy, distinguishing pre-specified from exploratory | Page 6. full statistical analysis, ROC curves, multivariable logistic regression |
|  |  | **18** | Intended sample size and how it was determined | Page 6. sample size calculation (n=345) |
|  | **RESULTS** |  |  |  |
|  | *Participants* | **19** | Flow of participants, using a diagram | Flowchart S-Figure 2; sample handling described in Methods |
|  |  | **20** | Baseline demographic and clinical characteristics of participants | Table 1; page 6-7 |
|  |  | **21a** | Distribution of severity of disease in those with the target condition | Table 1; S-Table 4; page 6-7 |
|  |  | **21b** | Distribution of alternative diagnoses in those without the target condition | Table 1; S-Table 4; page 6-7 |
|  |  | **22** | Time interval and any clinical interventions between index test and reference standard | Page 4-5, both obtained within first 24 h; SeptiScore at admission |
|  | *Test results* | **23** | Cross tabulation of the index test results (or their distribution)  by the results of the reference standard | Figure 1; Table 1; SeptiScore bands; page 6-8 |
|  |  | **24** | Estimates of diagnostic accuracy and their precision (such as 95% confidence intervals) | Table 2; Figures 2A–2B; S-Tables 7–8; page 6-8. |
|  |  | **25** | Any adverse events from performing the index test or the reference standard | N/A |
|  | **DISCUSSION** |  |  |  |
|  |  | **26** | Study limitations, including sources of potential bias, statistical uncertainty, and generalisability | Page 11, limitations section |
|  |  | **27** | Implications for practice, including the intended use and clinical role of the index test | Page 11, final paragraphs |
|  | **OTHER INFORMATION** |  |  |  |
|  |  | **28** | Registration number and name of registry | N/A |
|  |  | **29** | Where the full study protocol can be accessed | Technical details in S-Methods |
|  |  | **30** | Sources of funding and other support; role of funders | Page 2 “Supported by an unrestricted grant from Biocartis” |
|  |  |  |  |  |

Adapted from Bossuyt PM, et al. Clin Chem. 2015 Dec;61(12):1446-52

Supplemental Table 2. Final diagnoses in patients with ~~sterile inflammation~~ non-infectious critical illness.

| **Diagnoses** | **n** |
| --- | --- |
| Acute respiratory failure | 17 |
| Heart failure | 17 |
| Metabolic disorders | 9 |
| Hypovolemic shock | 7 |
| COPD exacerbation | 6 |
| Postoperative SIRS | 6 |
| Neurological disorders | 5 |
| Drug toxicity | 5 |
| Acute pancreatitis | 3 |
| Other causes ~~sterile inflammation~~ non-infectious critical illness | 11 |

SIRS: Systemic inflammatory response syndrome; COPD: Chronic obstructive Pulmonary disease

**Supplemental Table 3**. Final diagnoses in patients without sepsis but Septiscore Band 4

| **Diagnoses** | **n (%)** |
| --- | --- |
| Heart failure | 3 |
| Acute Pancreatitis | 2 |
| Cardiogenic shock | 2 |
| Postoperative SIRS | 2 |
| Acute renal failure | 2 |
| Acute leukaemia | 1 |
| COPD exacerbation | 1 |
| Haemorrhagic shock | 1 |
| Pulmonary aspiration | 1 |
| Pulmonary embolism | 1 |
| Pulmonary Hypertension | 1 |
| Unknown origin SIRS | 1 |

*COPD: Chronic obstructive pulmonary disease; SRIS: Systemic inflammatory response syndrome

Supplemental Table 4. Analysis of laboratory data differences between patients with sepsis/~~sterile inflammation~~ non-infectious critical illness established at ICU discharge.

| **Variable*** | **All patients**  **(n=353)** | **Sepsis**  **(n=267)** | **Non-infectious critical illness**  **(n=86)** | **p** |
| --- | --- | --- | --- | --- |
| Leucocytes cells/µl | 12.4 (7.3-18.7) | 12.2 (6.5 – 19.0) | 12.9 (10.1 – 17.4) | 0.085 |
| Neutrophils % WBC | 10.6 (6.0-15.5) | 10.4 (5.0 - 15.8) | 10.9 (7.6 - 14.5) | 0.156 |
| Platelets cells/µl | 198 (117-298) | 181 (110 - 277) | 256 (177 - 333) | <0.001 |
| Creatinine mg/dL | 1.50 (0.91-2.52) | 1.50 (0.96 - 2.40) | 1.36 (0.87 - 2.72) | 0.950 |
| Lactate mmol/L | 1.80 (1.00-3.20) | 2 (1.2 - 3.3) | 1.2 (0.8 - 2.8) | 0.001 |
| Procalcitonin ng/mL | 4.08 (0.54-24.0) | 9.2 (1.2 – 35.0) | 0.4 (0.1 – 1.6) | <0.001 |
| C Reactive protein mg/L | 171.6 (86.1-290.4) | 211.5 (122.7 - 307.4) | 82.0 (22.7 – 170.7) | <0.001 |

*Result expressed with median (p25-p75) or number (percentage) as appropriate.

Supplemental Table 5. Micro-organisms isolated in blood cultures.

|  | **n** | **%** |
| --- | --- | --- |
| *Acinetobacter baumannii* | 2 | 0.6% |
| *Bacillus* spp | 1 | 0.3% |
| *Bacteroides* grupo *fragilis* | 1 | 0.3% |
| *Candida albicans* | 3 | 0.8% |
| *Candida glabrata* | 1 | 0.3% |
| *Candida* spp | 1 | 0.3% |
| *Enterobacter cloacae* | 1 | 0.3% |
| *Enterobacter* spp | 1 | 0.3% |
| *Enterococcus faecalis* | 5 | 1.4% |
| *Enterococcus faecium* | 1 | 0.3% |
| *Escherichia coli* | 38 | 10.8% |
| *Klebsiella pneumoniae* | 14 | 4.0% |
| *Morganella morgagnii* | 1 | 0.3% |
| *Proteus mirabilis* | 4 | 1.1% |
| *Proteus* spp | 1 | 0.3% |
| *Pseudomonas aeruginosa* | 2 | 0.6% |
| *Salmonella* spp | 2 | 0.6% |
| Methicillin-sensitive *Staphylococcus aureus* | 5 | 1.4% |
| Methicillin-resistant *Staphylococcus aureus* | 4 | 1.1% |
| *Coagulase negative Staphylococcus* | 5 | 1.4% |
| Staphylococcus epidermidis | 3 | 0.8% |
| *Staphylococcus* spp | 1 | 0.3% |
| *Streptococcus pneumoniae* | 13 | 3.7% |
| *Streptococcus pyogenes* | 5 | 1.4% |
| *Streptococcus* spp | 7 | 2.0% |
| Other microorganisms* | 6 | 1.7% |

* *Aeromonas veronii*; *Fusobacterium necrophorum*; *Klebsiella aerogenes*; Gramnegative bacillus.

Supplemental Table 6. Micro-organisms isolated in source of infection.

|  | **n** | **%** |
| --- | --- | --- |
| *Acinetobacter baumannii* | 2 | 0.6% |
| *Aspergillus* spp | 4 | 1.1% |
| *Candida albicans* | 9 | 2.5% |
| *Citrobacter* spp | 1 | 0.3% |
| *Chlamydophila pneumoniae* | 2 | 0.6% |
| *Clostridium* spp | 2 | 0.6% |
| *Enterobacter cloacae* | 4 | 1.1% |
| *Enterobacter* spp | 1 | 0.3% |
| *Enterococcus faecalis* | 8 | 2.3% |
| *Enterococcus faecium* | 11 | 3.1% |
| *Enterococcus* spp | 1 | 0.3% |
| *Escherichia coli* | 37 | 10.5% |
| *Haemophilus influenzae* | 3 | 0.8% |
| *Haemophilus* spp | 1 | 0.3% |
| *Klebsiella oxytoca* | 2 | 0.6% |
| *Klebsiella pneumoniae* | 15 | 4.2% |
| *Klebsiella* spp | 1 | 0.3% |
| *Legionella pneumophila* | 2 | 0.6% |
| *Mycobacterium tuberculosis* | 1 | 0.3% |
| *Proteus mirabilis* | 6 | 1.7% |
| *Pseudomonas aeruginosa* | 9 | 2.5% |
| *Salmonella* spp | 2 | 0.6% |
| Methicillin-sensitive *Staphylococcus aureus* | 7 | 2.0% |
| Methicillin-resistant *Staphylococcus aureus* | 4 | 1.1% |
| *Coagulase negative Staphylococcus* | 4 | 1.1% |
| Staphylococcus epidermidis | 1 | 0.3% |
| *Stenotrophomonas maltophilia* | 3 | 0.8% |
| *Streptococcus pneumoniae* | 23 | 6.5% |
| *Streptococcus pyogenes* | 3 | 0.8% |
| *Streptococcus* spp | 11 | 3.1% |
| HVS virus | 1 | 0.3% |
| Influenza virus | 3 | 0.8% |
| SARS-CoV-2 virus | 6 | 1.7% |
| Other microorganisms* | 27 | 7.6% |

* *Enteroviridae;* Aerococcus urinae*;* *Bacteroides faecalis*; *Fusobacterium nucleatum*; *Parabacteroides distanosis;* *Plasmodium falciparum;* *Veillonella;* Rinovirus; HVS virus 1+2; *Gramnegative* bacillus.

Supplemental Table 7. Cut-off point of maximum sensitivity and specificity of Septiscore.

|  | **Septiscore ≥ 6.9** | | |  | **For sepsis** | | CI 95% |
| --- | --- | --- | --- | --- | --- | --- | --- |
|  | SEPSIS | ~~STERILE INFLAMMATION~~  NON-INFECTIOUS CRITICAL ILLNESS |  |  | Se | 85.4% | 80.5%-89.4% |
| YES | 228 | 20 | 248 |  | Sp | 76.7% | 65.5%-84.4% |
| NO | 39 | 66 | 105 |  | PPV | 91.9% | 88.2%-94.0% |
|  | 267 | 86 | 353 |  | NPV | 62.9% | 55.2%-69.8% |
|  |  |  |  |  | LR+ | 3.67 | 2.43-5.15 |
|  |  |  |  |  | LR- | 0.19 | 0.14-0.26 |
|  |  |  |  |  | AC | 83.3% | 78.7%-86.8% |
|  |  |  |  |  | PREV | 75.6% | 70.5%-80.0% |

Supplemental Table 8. Cut-off point of maximum sensitivity and specificity of Septiscore combined with Procalcitonin or C-reactive protein cut-off points (Youden test)

|  | **Sensitivity(CI95%)** | **Specificity(CI95%)** | **PPV(CI95%)** | **NPV(CI95%)** | **LR +(CI95%)** | **LR -(CI95%)** | **AC(CI95%)** |
| --- | --- | --- | --- | --- | --- | --- | --- |
| **PCT≥2.8 + SEPTISCORE ≥6.9** | 61.0%(54.9%-66.9%) | 91.9%(83.9%-96.7%) | 95.9%(91.9%-97.9%) | 43.2%(39.2%-47.2%) | 7.50(3.66-15.35) | 0.42(0.36-0.50) | 82.7%(78.4%-86.5%) |
| **CRP ≥ 150 + SEPTISCORE ≥6.9** | 62.5%(56.4%-68.3%) | 83.7%(74.2%-90.8%) | 92.3%(88.0%-95.1%) | 41.9%(37.6%-46.4%) | 3.84(2.36-6.26) | 0.45(0.37-0.54) | 77.1%(72.3%-81.3%) |

AC: Accuracy; LR: Likelihood ratio; NPV: Negative predictive value; PPV: Positive predictive value.

Supplemental Table 9. Correlation between Septiscore and other markers and scores

| **Variable** | |  | | **Septiscore** | |
| --- | --- | --- | --- | --- | --- |
| 1. Septiscore |  | Pearson's r |  | — |  |
|  |  | p-value |  | — |  |
| 2. Log PCT |  | Pearson's r |  | 0.489 | *** |
|  |  | p-value |  | < .001 |  |
| 3. CRP |  | Pearson's r |  | 0.612 | *** |
|  |  | p-value |  | < .001 |  |
| 4. Age |  | Pearson's r |  | -0.163 | ** |
|  |  | p-value |  | 0.002 |  |
| 5. BMI |  | Pearson's r |  | -0.106 | * |
|  |  | p-value |  | 0.047 |  |
| 6. APACHEII |  | Pearson's r |  | -0.055 |  |
|  |  | p-value |  | 0.307 |  |
| 7. SOFA |  | Pearson's r |  | -0.012 |  |
|  |  | p-value |  | 0.818 |  |

* p < .05, ** p < .01, *** p < .001

APACHE II: Acute Physiology and Chronic Health disease Classification System II; BMI: Body Mass Index; ~~CNS: Central nervous system~~; CRP: C reactive protein; PCT: Procalcitonin; SOFA: Sequential Organ Failure Assessment Score.

Supplemental Table 10. Demographics, clinical and analytical characteristics of patients with sepsis and Band 1 and non-infectious critical illness and Band 4 established at ICU discharge.

| **Variable*** | | **All patients**  **(n=353)** | **Sepsis and Band 1**  **(n=9)** | **Non-infectious critical illness** **and Band 4**  **(n=18)** |  |
| --- | --- | --- | --- | --- | --- |
| Age | | 63 (54 - 71) | 66 (60 - 76) | 54 (47 - 60) | 0.023 |
| Gender | Male | 219 (62.0%) | 6 (66.7%) | 15 (83.3%) | 0.326 |
| BMI | | 27.3 (24.2-32.0) | 26.8 (24.2 – 28.4) | 23.8 (22.2 – 26.3) | 0.375 |
| Comorbidities | Diabetes mellitus | 117 (33.1%) | 2 (22.2%) | 3 (16.7%) | 0.553 |
|  | Renal insufficiency | 49 (13.9%) | 1 (11.1%) | 2 (11.1%) | 0.750 |
|  | Immunosuppression | 47 (13.3%) | 0 (0%) | 3 (16.7%) | 0.279 |
|  | Neutropenia | 8 (2.3%) | 0 (0%) | 0 (0%) | NA |
|  | Solid neoplasm | 61 (17.3%) | 2 (22.2%) | 1 (5.6%) | 0.250 |
|  | Hepatic cirrhosis | 17 (4.8%) | 0 (0%) | 0 (0%) | NA |
|  | COPD | 48 (13.6%) | 1 (11.1%) | 3 (16.7%) | 0.593 |
|  | Cardiac insufficiency | 23 (6.5%) | 0 (0%) | 4 (22.2%) | 0.174 |
|  | Autoimmune diseases | 18 (5.1%) | 0 (0%) | 0 (0%) | NA |
|  | SARS - CoV2 infection | 17 (4.8%) | 0 (0%) | 0 (0%) | NA |
|  | None of previous | 124 (35.1%) | 6 (66.7%) | 7 (38.9%) | 0.171 |
| Type of ICU admission | Medical condition | 280 (79.3%) | 9 (100%) | 15 (83.3%) | 0.430 |
|  | Scheduled surgery | 8 (2.3%) | 0 (0%) | 1 (5.6%) |  |
|  | Urgent surgery | 65 (18.4%) | 0 (0%) | 2 (11.1%) |  |
| Acquisition of infection | Community | 267 (75.6%) | 5 (55.6%) | 16 (88.9%) | 0.073 |
|  | Nosocomial | 86 (24.4%) | 4 (44.4%) | 2 (11.1%) |  |
| Heart rate | | 110 (90-120) | 92 (80 - 110) | 100 (85 - 120) | 0.275 |
| Respiratory rate | | 22 (18-28) | 22 (18 - 26) | 24 (18 - 28) | 0.561 |
| Mean arterial pressure | | 64 (54-77) | 60 (49 - 85) | 76 (70 - 92) | 0.059 |
| APACHE II score | | 18 (14 – 24) | 21 (17 - 23) | 16 (9 - 25) | 0.253 |
| SOFA score | | 7 (5 - 9) | 7 (6 - 10) | 5 (3 - 8) | 0.076 |
| Leucocytes | | 12.4 (7.3-18.7) | 12.6 (9.7 – 21.4) | 11.8 (10.1 – 17.4) | 0.900 |
| Neutrophils | | 10.6 (6.0-15.5) | 9.9 (7.5 – 17.3) | 9.5 (7.5 - 14.5) | 0.860 |
| Platelets | | 198 (117-298) | 225 (145 - 302) | 209 (168 - 326) | 0.940 |
| Creatinine | | 1.50 (0.91-2.52) | 1.64 (1.28 - 2.16) | 0.98 (0.74 – 2.71) | 0.322 |
| Lactate | | 1.80 (1.00-3.20) | 2.4 (0.5 - 3.5) | 1.2 (0.7 – 2.7) | 0.348 |
| Procalcitonin | | 4.08 (0.54-24.0) | 0.7 (0.6 – 7.3) | 1.2 (0.2 – 15.1) | 0.940 |
| C-Reactive protein | | 171.3 (85.2-290.0) | 91.2 (61.9 – 113.0) | 227.2 (140.4 – 323.6) | 0.005 |
| Septiscore | | 8.4 (6.5-10.1) | 4.1 (3.7 – 4.6) | 9.2 (8.4 – 10.2) | <0.001 |
| Band of Septiscore | BAND 1 (0-4.9) | 35 (9.9%) | 9 (100%) | 0 (0%) | NA |
|  | BAND 2 (5.0-6.1) | 40 (11.3%) | 0 (0%) | 0 (0%) |  |
|  | BAND 3 (6.2-7.3) | 48 (13.6%) | 0 (0%) | 0 (0%) |  |
|  | BAND 4 (7.4-15) | 230 (65.2%) | 0 (0%) | 18 (100%) |  |
| Shock | No shock | 131 (37.1%) | 1 (11.1%) | 8 (44.4%) | 0.094 |
|  | Shock | 222 (62.9%) | 8 (88.9%) | 11 (55.6%) |  |
| Physicians subjective likelihood of sepsis at ICU admission | < 30% | 40 (11.3%) | 0 (0%) | 5 (27.8%) | 0.022 |
|  | 30-70% | 97 (27.5%) | 3 (33.3%) | 10 (55.6%) |  |
|  | >70% | 216 (61.2%) | 6 (66.7%) | 3 (16.7%) |  |
| Blood culture at admission | Positive | 105 (29.7%) | 1 (11.1%) | 0 ** | 0.617 |
| Suspected Source | Abdominal | 116 (32.9%) | 1 (11.1%) | 4 (22.2%) | 0.447 |
|  | Bacteremia | 19 (5.4%) | 0 (0.0%) | 1 (5.6%) | 0.667 |
|  | Cardiovascular | 7 (2.0%) | 0 (0.0%) | 2 (11.1%) | 0.436 |
|  | Catheter related | 5 (1.4%) | 0 (0.0%) | 0 (0.0%) | NA |
|  | Gynaecological | 4 (1.1%) | 0 (0.0%) | 0 (0.0%) | NA |
|  | Surgical wound | 2 (0.6%) | 0 (0.0%) | 1 (5.6%) | 0.667 |
|  | Ophthalmologic | 0 (0.0%) | 0 (0.0%) | 0 (0.0%) | NA |
|  | Ear, nose and throat | 3 (0.8%) | 0 (0.0%) | 0 (0.0%) | NA |
|  | Bones | 2 (0.6%) | 0 (0.0%) | 0 (0.0%) | NA |
|  | Other foci | 3 (0.8%) | 0 (0.0%) | 0 (0.0%) | NA |
|  | Unknown source | 12 (3.4%) | 0 (0.0%) | 0 (0.0%) | NA |
|  | Soft tissue | 13 (3.7%) | 0 (0.0%) | 0 (0.0%) | NA |
|  | Respiratory | 110 (31.2%) | 4 (44.4%) | 9 (50.0%) | 0.555 |
|  | CNS | 19 (5.4%) | 0 (0.0%) | 0 (0.0%) | NA |
|  | Urological | 38 (10.8%) | 4 (44.4%) | 1 (5.6%) | 0.030 |
| RRT first 72 h | Yes | 77 (21.8%) | 2 (22.2%) | 6 (33.3%) | 0.450 |
| IMV first 72 h | Yes | 186 (52.7%) | 5 (55.6%) | 10 (55.6%) | 0.657 |
| Vasopressors first 72 h | Yes | 228 (64.6%) | 9 (100%) | 6 (33.3%) | 0.001 |
| ICU stays (days) | | 5 (2 – 12) | 8 (5 - 12) | 3 (1 - 13) | 0.176 |
| Hospital stays (days) | | 16 (8 – 35) | 17 (13 - 42) | 15 (8 - 24) | 0.194 |
| ICU death | Yes | 76 (21.5%) | 2 (22.2%) | 2 (11.1%) | 0.407 |
| Hospital death | Yes | 93 (26.4%) | 2 (22.2%) | 2 (11.1%) | 0.407 |

*Result expressed with median (p25-p75) or number (percentage) as appropriate.

** 1 coagulase-negative Staphylococci, considered contaminant by the researches.

APACHE II: Acute Physiology and Chronic Health disease Classification System II; BMI: Body Mass Index; CNS: Central nervous system; COPD: Chronic obstructive pulmonary disease; ICU: Intensive Care Unit; IMV: Invasive mechanical ventilation; RRT: Renal replacement therapy; SOFA: Sequential Organ Failure Assessment Score.

Supplemental Figure 1. Sepsis Probability Bands (according to the manufacturer).


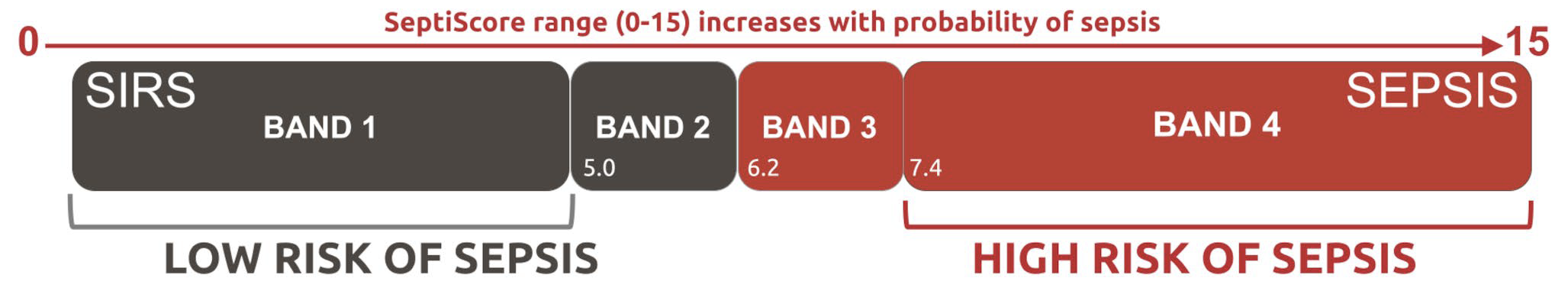


Supplemental Figure 2. Flowchart of the patients included in this study.

**
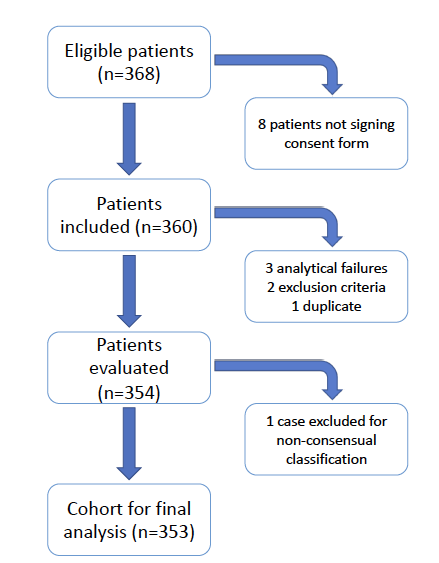
**

Supplemental Figure 3. Number of valid cases in every hospital.


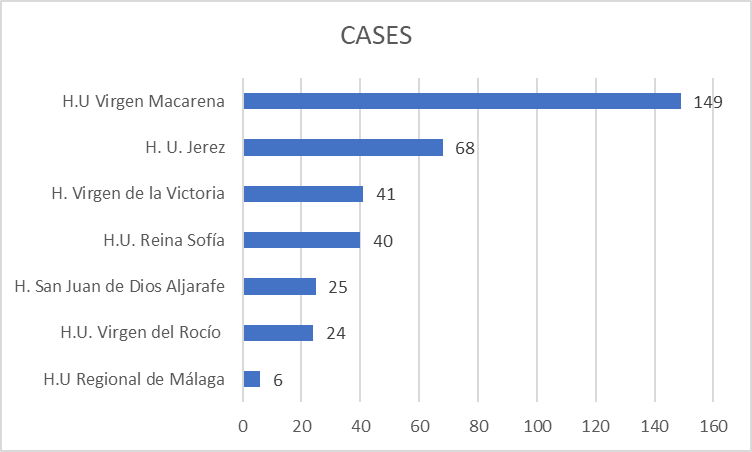


Supplemental Figure 4. Clinical performance of the 4 bands for the diagnosis of sepsis.

Supplemental Figure 5. Fagan nomogram for Septiscore BAND 4.


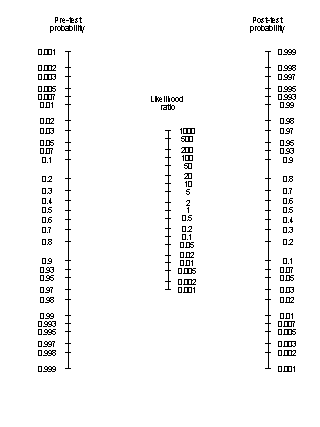


Supplemental Figure 6. Comparation of Septiscore values between definitely septic patients with sepsis or septic shock. Boxplots and violin plots.


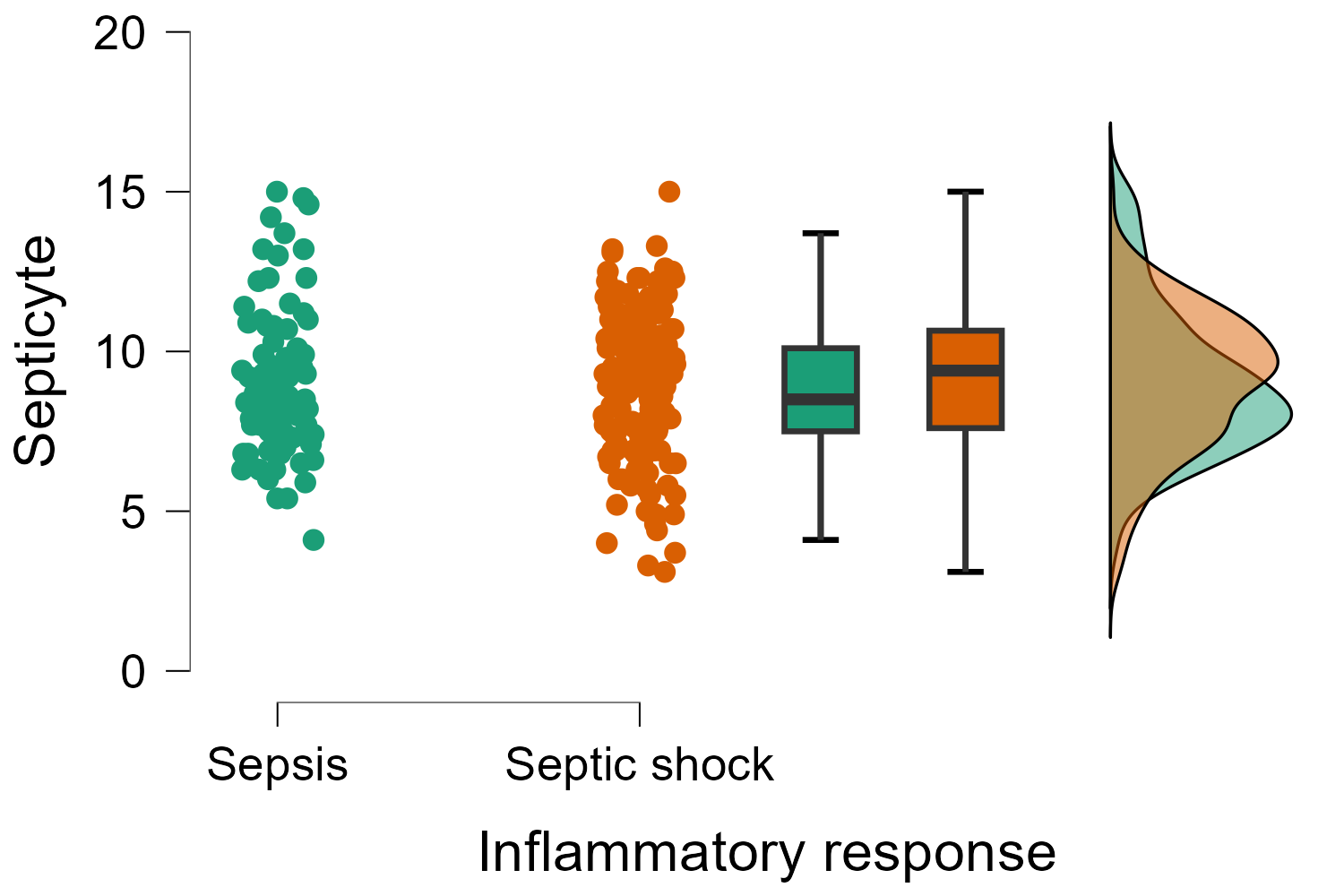


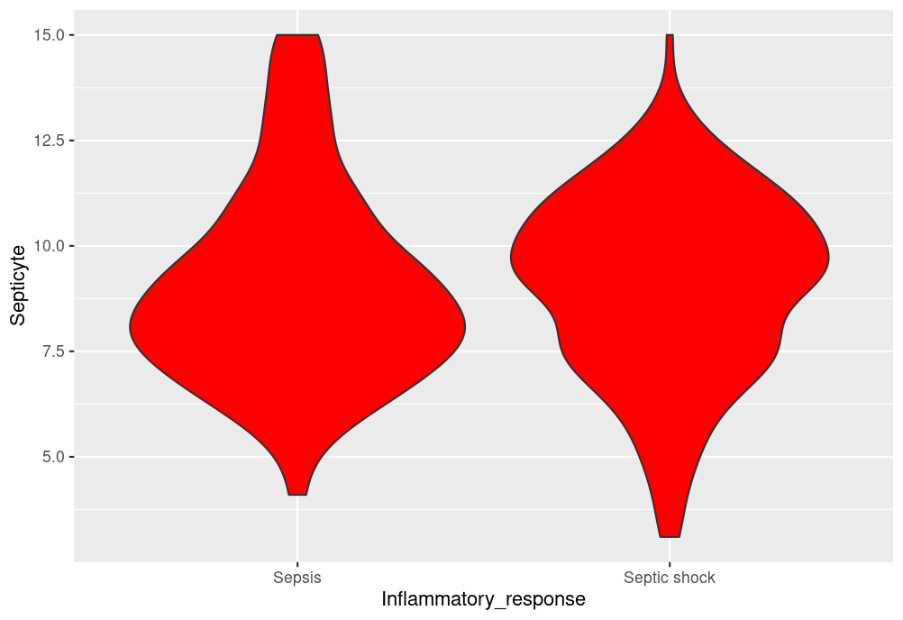


Supplemental Figure 7. Comparation of Septiscore values between definitely septic patients bacteremic and not bacteremic. Boxplots and violin plots.


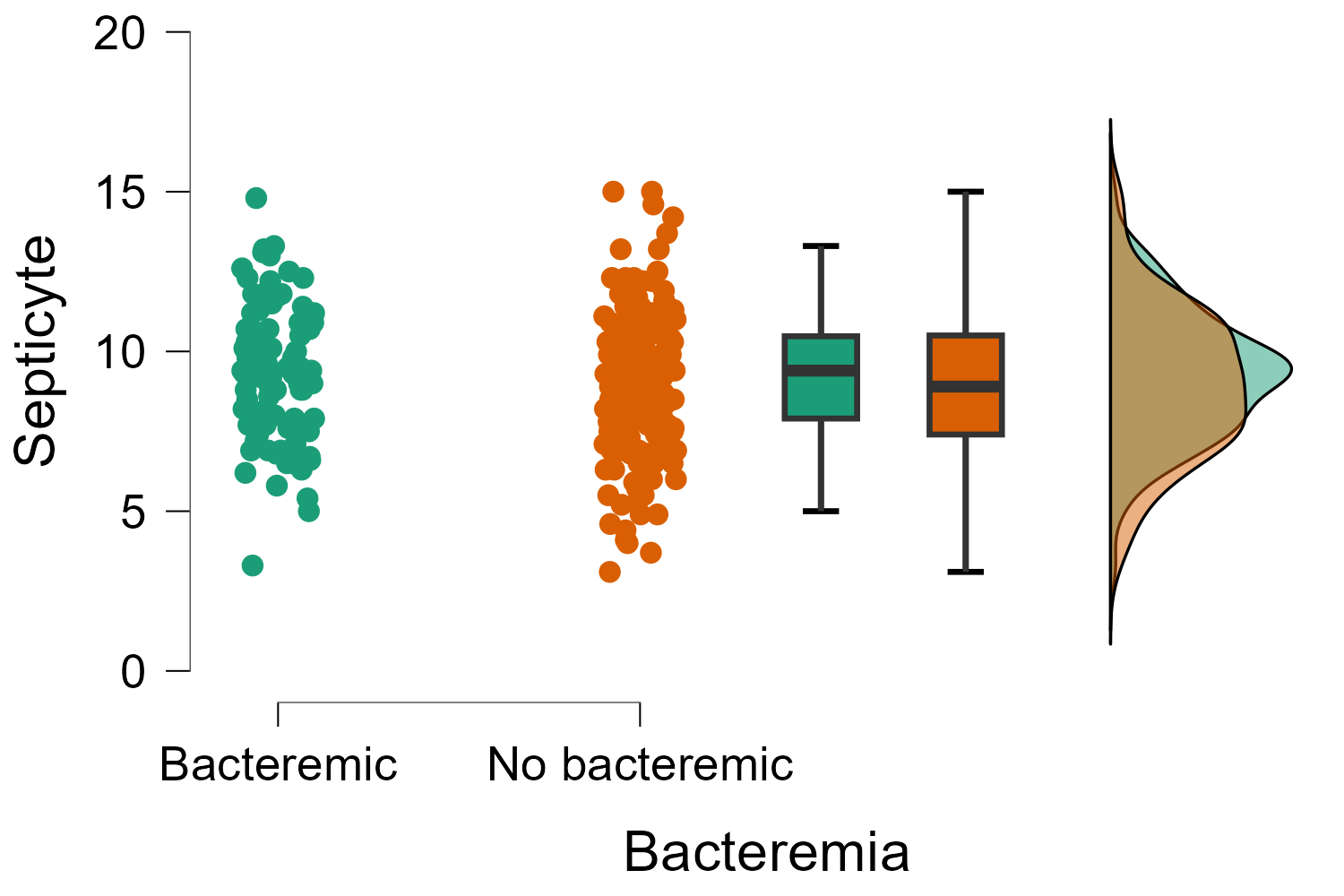


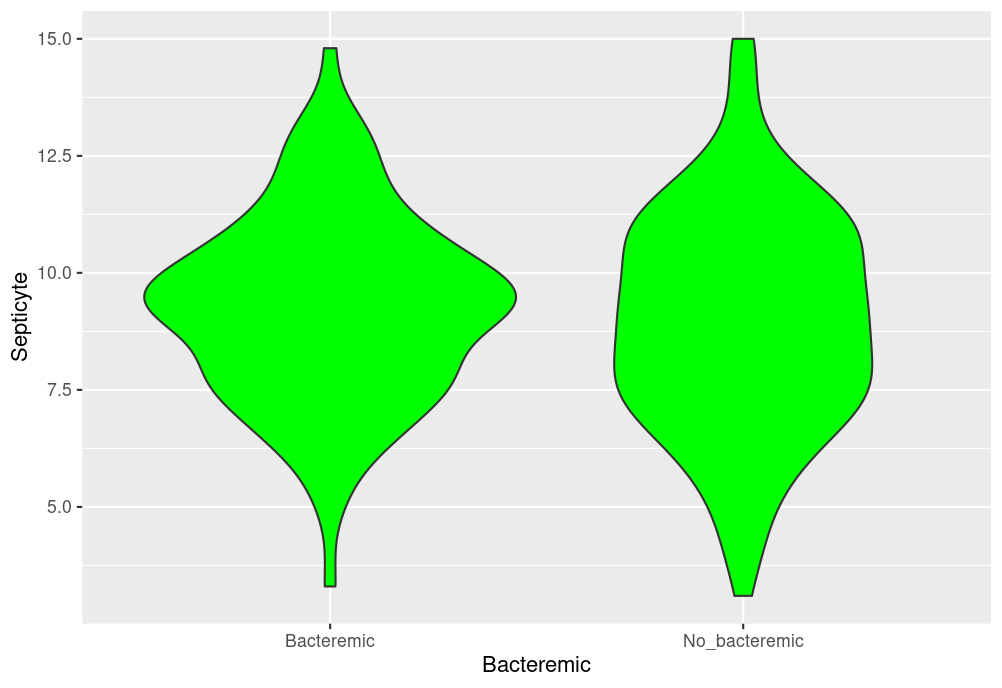


Supplemental Figure 8. Curves for SeptiScore, PCT, CRP and results of the pairwise AUROC comparisons.


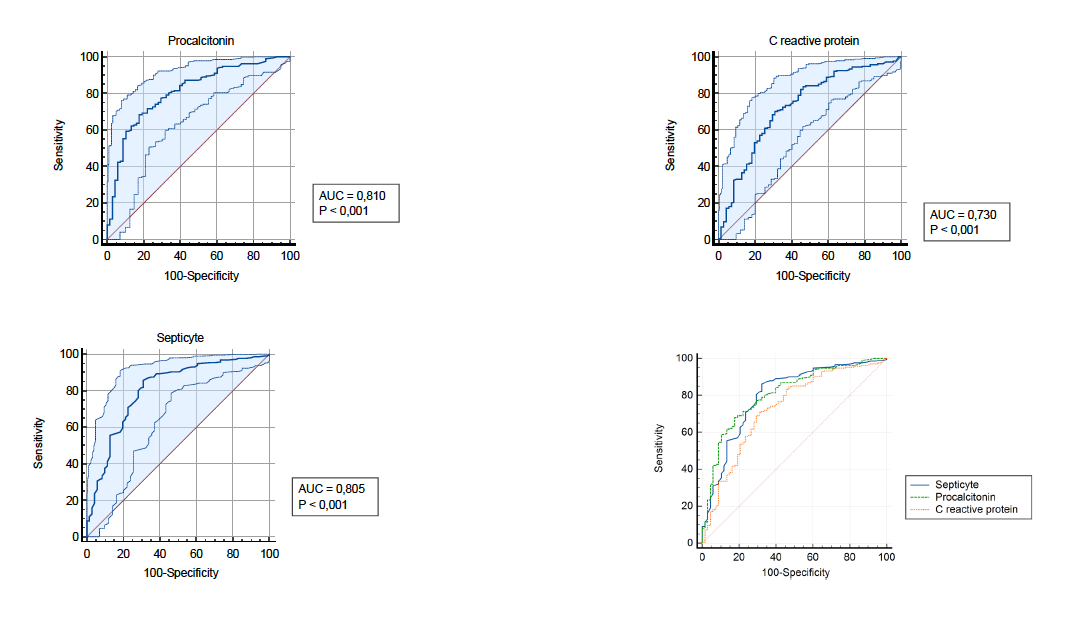


| Septicyte ~ Procalcitonin | |
| --- | --- |
| Difference between areas | 0,00802 |
| Standard Error ^a^ | 0,0357 |
| 95% Confidence Interval | -0,0619 to 0,0780 |
| z statistic | 0,225 |
| Significance level | P = 0,8222 |
| Septicyte ~ C reactive protein | |
| Difference between areas | 0,0697 |
| Standard Error ^a^ | 0,0321 |
| 95% Confidence Interval | 0,00670 to 0,133 |
| z statistic | 2,168 |
| Significance level | P = 0,0301 |
| Procalcitonin ~ C reactive protein | |
| Difference between areas | 0,0777 |
| Standard Error ^a^ | 0,0360 |
| 95% Confidence Interval | 0,00720 to 0,148 |
| z statistic | 2,160 |
| Significance level | P = 0,0308 |

Supplemental Figure 9. Comparation of models to predict Sepsis.


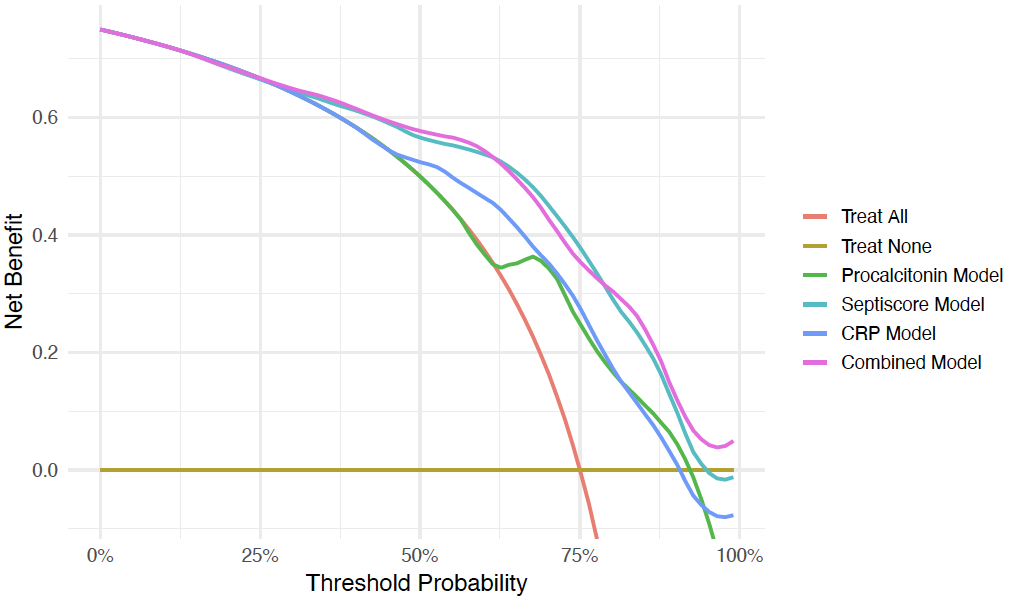


Supplemental Figure 10. Performance of Septicyte and PCT as predictors of in-hospital mortality


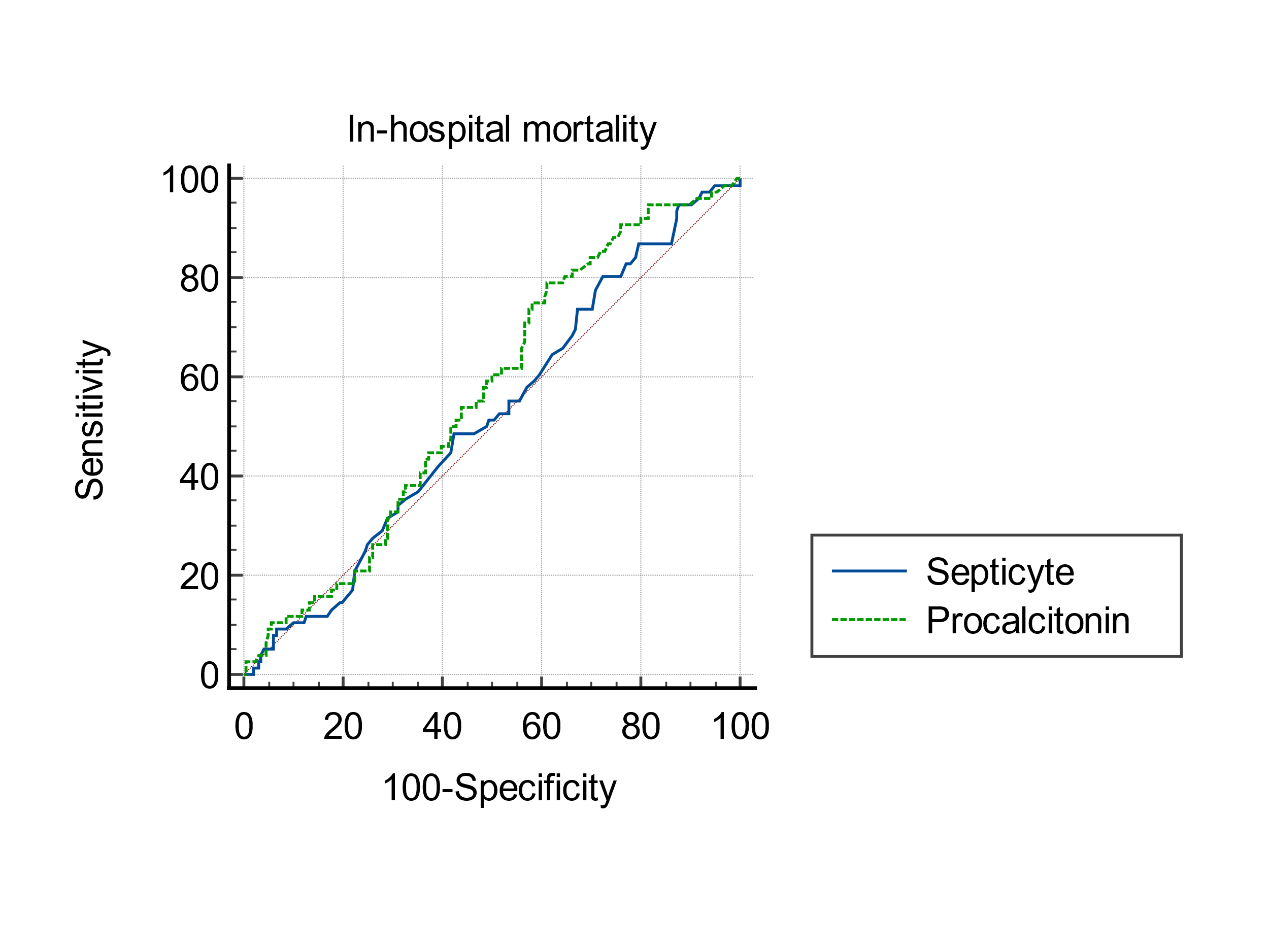


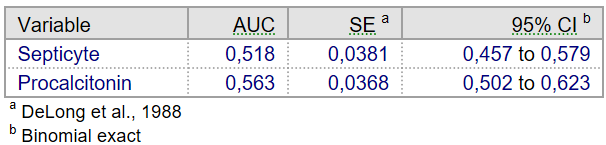


Supplemental Figure 11. Performance of Septicyte and PCT as predictors of ICU mortality


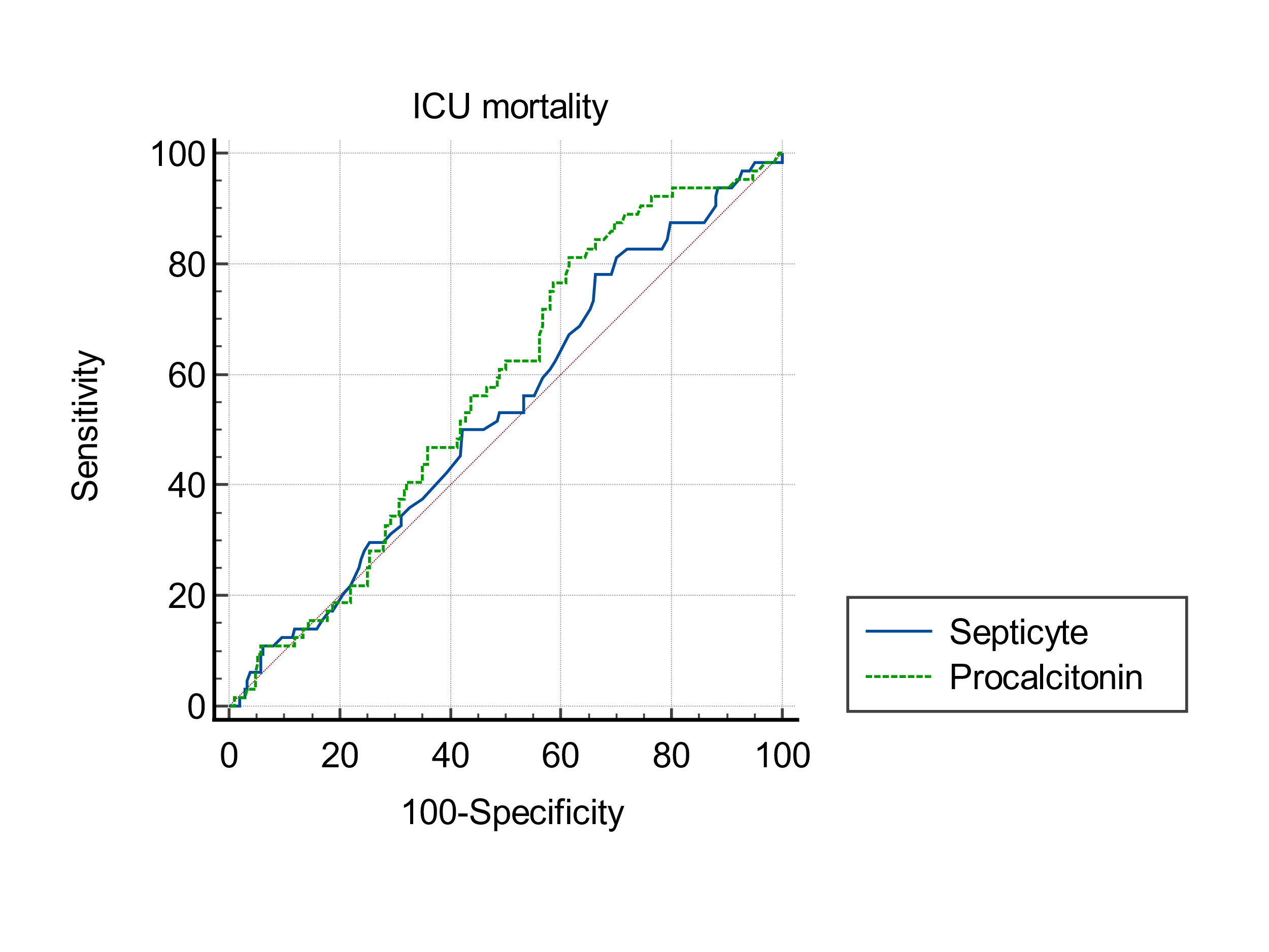


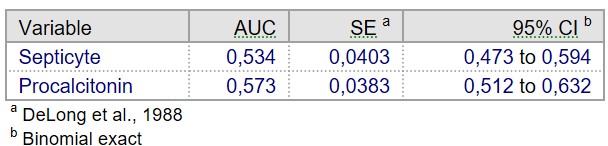


Supplemental Figure 12. Correlation between Septiscore and other markers and scores


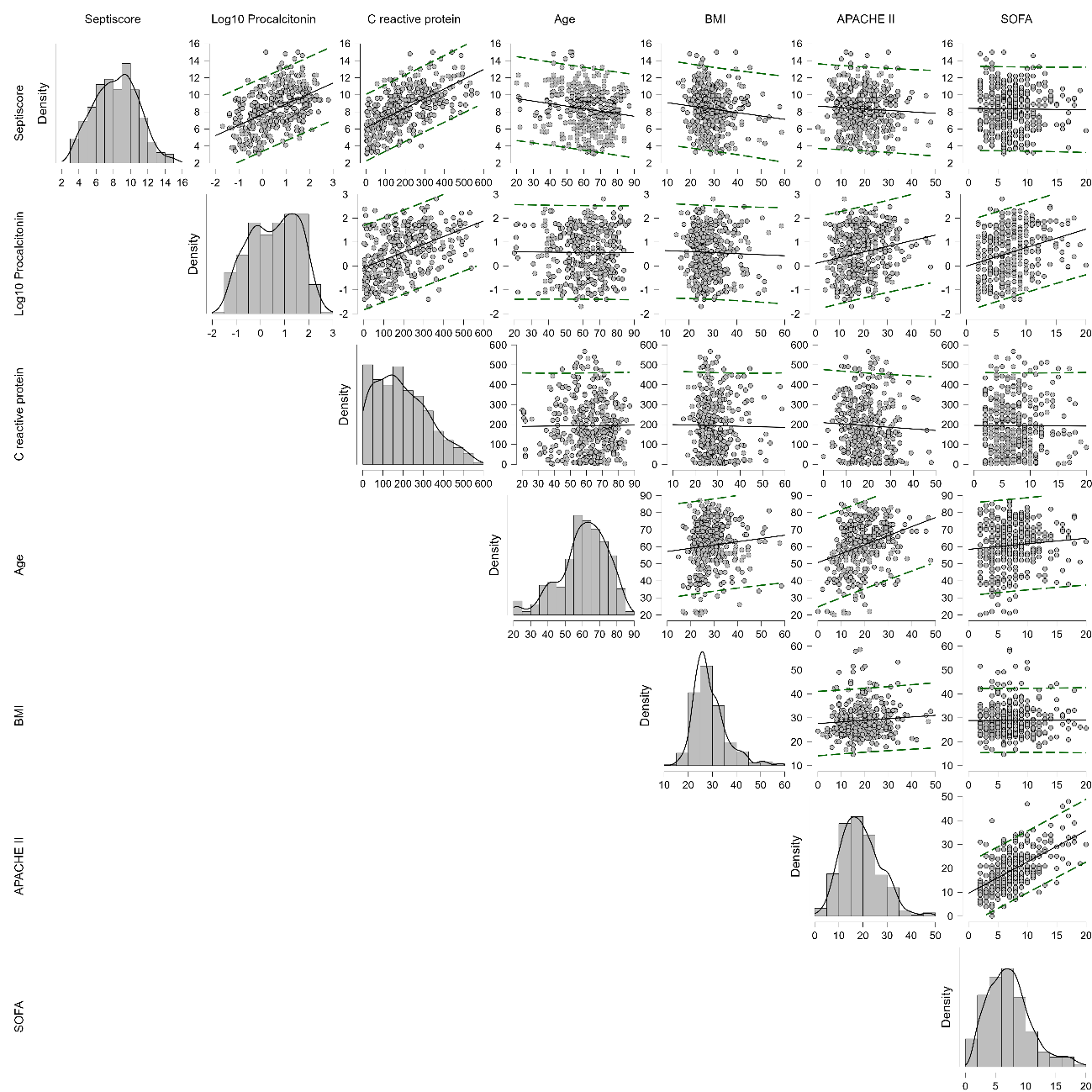

Supplement: Supplementary file 1 — Supplementary Material 1 (DOCX 4.89 MB) [file 10096_2026_5431_MOESM1_ESM.docx]
